# Glycoscience@Synchrotron: Synchrotron radiation applied to structural glycoscience

**DOI:** 10.3762/bjoc.13.114

**Published:** 2017-06-14

**Authors:** Serge Pérez, Daniele de Sanctis

**Affiliations:** 1 Department of Molecular Pharmacochemistry, CNRS-University Grenoble Alpes, France; 2 ESRF – The European Synchrotron, Grenoble, France

**Keywords:** antibodies, carbohydrate binding domains, cellulose, glycosaminoglycans, glycolipids, glycosyl hydrolases, glycosyl transferases, kinetic crystallography, lectins, polysaccharides, powder diffraction, small-angle X-ray scattering, starch, synchrotron radiation, transporters, X-ray crystallography

## Abstract

Synchrotron radiation is the most versatile way to explore biological materials in different states: monocrystalline, polycrystalline, solution, colloids and multiscale architectures. Steady improvements in instrumentation have made synchrotrons the most flexible intense X-ray source. The wide range of applications of synchrotron radiation is commensurate with the structural diversity and complexity of the molecules and macromolecules that form the collection of substrates investigated by glycoscience. The present review illustrates how synchrotron-based experiments have contributed to our understanding in the field of structural glycobiology. Structural characterization of protein–carbohydrate interactions of the families of most glycan-interacting proteins (including glycosyl transferases and hydrolases, lectins, antibodies and GAG-binding proteins) are presented. Examples concerned with glycolipids and colloids are also covered as well as some dealing with the structures and multiscale architectures of polysaccharides. Insights into the kinetics of catalytic events observed in the crystalline state are also presented as well as some aspects of structure determination of protein in solution.

## Introduction

**Figure 1:**
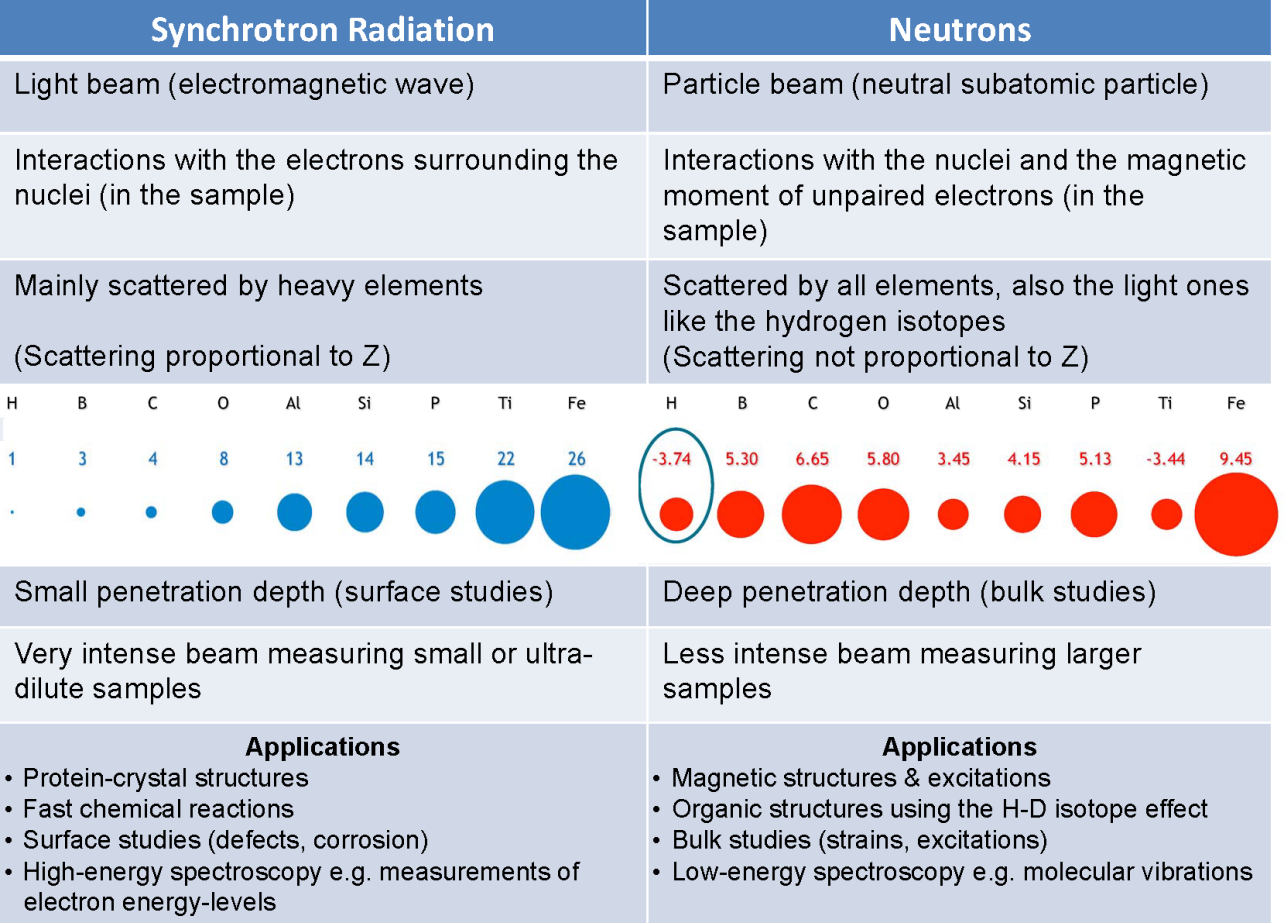
Complementarity of synchrotron radiation and neutron sources to investigate the structure of matter.

Over the last decade, glycoscience has greatly benefited from the development of structural biology and the investigation of macromolecular structure and function relationships. Major contributions also came from considerable advances in high resolution NMR spectrometry and electron microscopy along with the continuous evolution of synchrotron radiation and free electron laser light sources. Since its discovery, X-ray radiation has been an invaluable tool to investigate the structure of matter. The range of wavelengths, in the region of an angstrom, and energies, extending over electronic shell levels, make them the perfect probe to study material at the atomic scale. Nevertheless, the low availability and versatility of sources had for a long time represented a limitation on the use of X-rays for scientific applications. A major breakthrough came from the advent of synchrotron science. Over the years, they became an indispensable resource in the exploration of matter, thanks to the continuous spectrum of emitted radiation, the extremely high flux and brightness. Those features allow a wide range of experiments, spanning virtually all branches of sciences and technological applications, particularly those akin to nanoscience. Developments in neutron sources have paralleled those of synchrotron sources. Figure 1 summarizes the differences and the complementarity of the information that can be gathered from analyses performed with the respective sources. The synergistic use of both sources becomes particularly relevant when accurate hydrogen details are necessary.

Structural glycobiology gained recognition with the elucidation of glycosyl hydrolases mechanism by X-ray crystallography, but the scope of applications in glycobiology is much broader: it encompasses the range of glycan containing (macro)-molecules and their conjugates. The present article reviews the application of synchrotron radiation to some key areas of glycoscience potentially of interest to the growing number of non-specialist users.

Structural characterization of protein–carbohydrate interactions are covered as well as some involving glycolipids and colloids and the structure and architecture of polysaccharides. Insights into the kinetics of catalytic events occurring in the crystalline state are also described as well as some aspects of the determination of structure of proteins in solution.

## Review

### Synchrotron radiation

**Figure 2:**
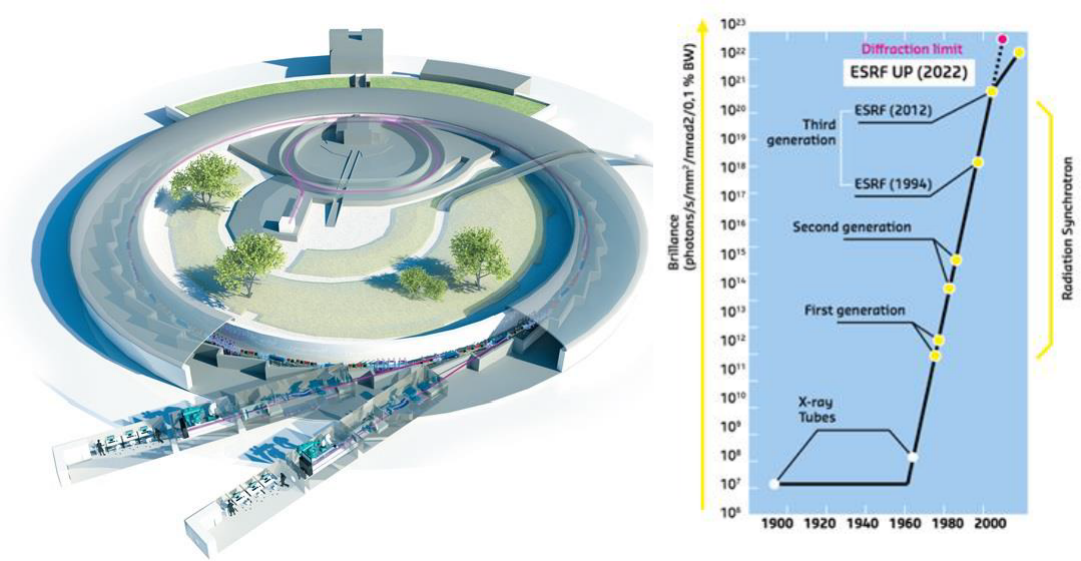
A representation of a synchrotron storage ring, including linear accelerator, booster and two beamlines (left) and the increase in X-ray brilliance since the first X-ray tubes to the current ESRF configuration and the predicted next generation after the machine upgrade planned in 2020. Credit: S. Gerlier/ESRF with permission.

**Figure 3:**
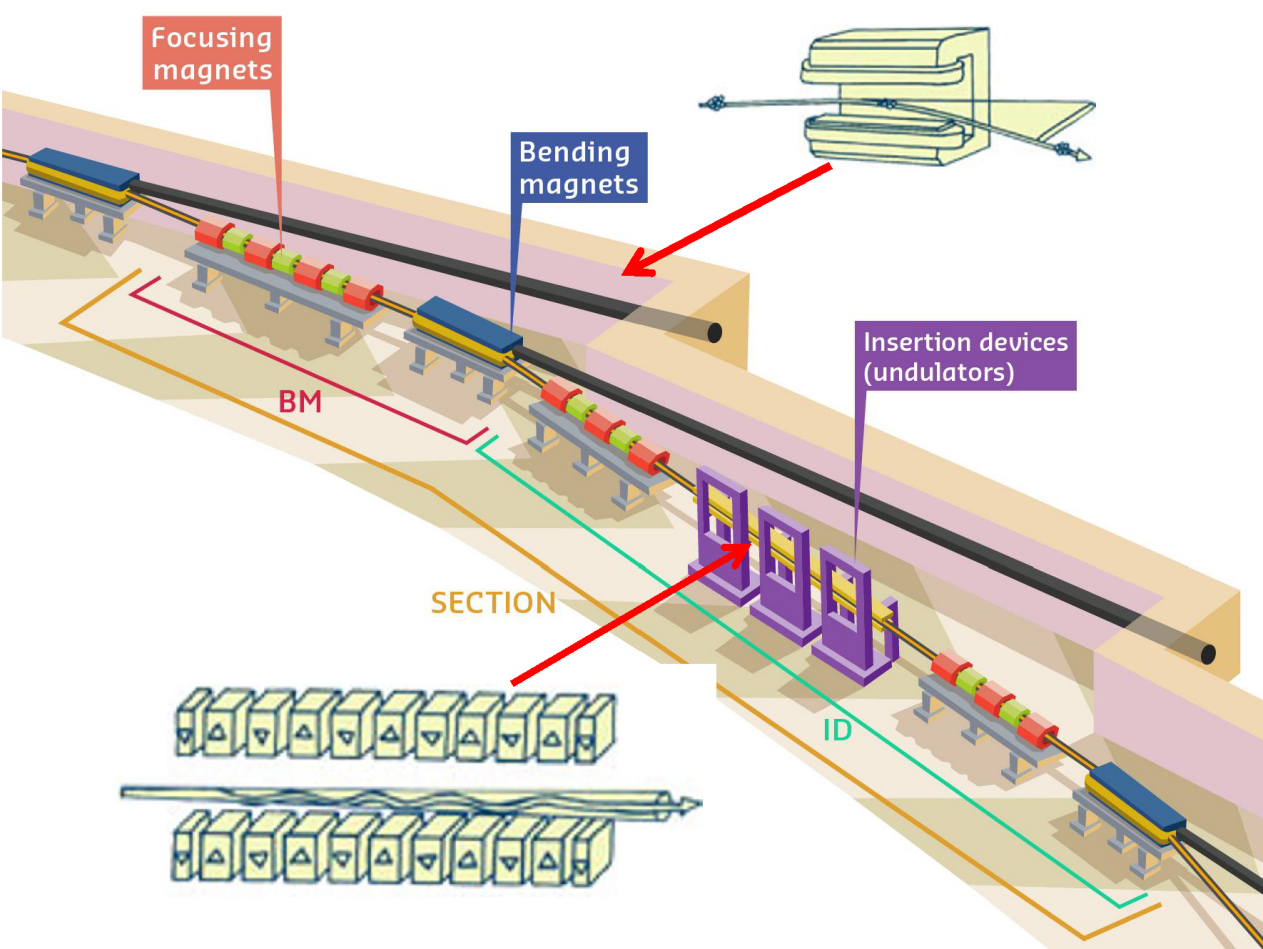
Schematic representation of a sector of a storage ring. Bending magnets and insertion devices are alternated. Bending magnets emit X-rays over a large angular range (top right) and are responsible for maintaining the closed trajectory in the storage ring. Insertion devices such as undulators (bottom left) produce X-rays with higher brilliance, which propagate along the electron beam. Credit: S. Gerlier/ESRF with permission.

Synchrotrons are particle accelerators in which charged particles circulate along a closed path. Storage rings are a particular kind of synchrotron in which the charged particles, usually electrons, are accelerated to speeds close to *c*, the speed of light, and kept orbiting at constant energy (Figure 2). In practice, the terms synchrotron and storage ring are often used interchangeably. The application of magnetic fields induces curvature in the trajectories of the particles, which lose energy by emitting electromagnetic radiation, known as synchrotron light. The electrons are forced to deviate from a straight trajectory either by bending magnets that present a constant dipolar magnetic field and ensure the closing of the orbit, or by insertion devices, such as undulators. Undulators are a much more efficient way to produce X-ray beams and force electrons along an oscillating path in the horizontal plane (Figure 3). In this manner, the X-ray emitted at one oscillation is in phase with the radiation from the following oscillations, resulting in an intrinsic higher brilliance. The improvements in insertion devices have made storage rings the most versatile intense X-ray sources, and many storage rings have been constructed around the world and planning for the construction and commissioning of a new generation of storage rings is under way [1].

In the quantum mechanics wave-particle duality, X-rays produced by a synchrotron can be regarded as a linearly polarized electromagnetic plane wave or as photons with energy given by Planck’s law. An X-ray photon that interacts with an atom can either be scattered or absorbed. Scattering that occurs with the same momentum (where there is no change in wavelength between scattered and incident waves) is called elastic or Thomson scattering. This is not generally the case, as an incident photon can transfer part of its energy to the electron and is scattered at a lower frequency by a phenomenon known as Compton scattering.

Photoelectric absorption occurs instead when an atom absorbs an X-ray photon. The excess energy is transferred to an electron, which is expelled and the atom is ionized. When the incident photon has an energy above the atomic K shell (so called K-edge energy), it expels an electron from the inner shell and creates a hole, which is eventually filled by an electron decaying from an outer shell. The difference in energy is emitted as a photon of energy equal to the difference of the two atomic shells. This effect is known as X-ray fluorescence and the photon energy provides a unique fingerprint of that atom. Moreover, modulation in absorption around the edge reflects the local structure of the material [2]. Photoelectric absorption, besides depending on the energy, varies with the Z atomic number (approximately proportional to Z^4^). This phenomenon produces the contrast that is used in X-ray imaging techniques.

The synchrotron light spectrum is polychromatic, with a spectral bandwidth that depends on the type and configuration of the sources. Many experiments require a narrower bandwidth, or a monochromatic beam, and this is produced using a perfect crystal, a monochromator, and the desired wavelength is selected by changing the angle of the incident beam, in accordance with Bragg’s law [3]. The monochromatic beam is then focused by using, for example, a system of X-ray mirrors. Consequently, an X-ray beam of the desired size and shape is delivered to the sample position. Continuous development of focusing systems has led to the use of beam sizes as small as a few nanometers. This has allowed the study of smaller samples with an enhanced signal to noise and higher spatial resolution [4–7].

The tunability of the wavelength to reach the values that are optimum for a given experiment provides the most powerful way to determine the three-dimensional features of macromolecular structures. Diffraction performed at an energy close to a heavy element absorption edge produces a resonant effect for which scattered waves are reemitted with a phase delay, inducing small variations in the diffraction intensities. The differences in the intensities can be used to determine the position of the heavier atoms and ultimately the electron density map of the macromolecule’s structure. This effect is known as multiwavelength anomalous diffraction (MAD) and today, with its single-wavelength variant (SAD), it is the most successful and widely used techniques to determine the 3D structure of complex systems such as biological molecules, which can be composed of thousands of atoms [8–9].

### Molecular structures

As early as 1930, the first crystal structures of organic compounds to be investigated were carbohydrates of low molecular weight. Over the following years, only eight additional crystal structures were reported. The determination of the three dimensional structure of the dehydrated form of sucrose, in 1947, was considered a significant contribution to the field. A major breakthrough occurred in 1951, when Bijvoet confirmed, without ambiguity, the D-configuration of glucose, which had been assigned from indirect reasoning by Emil Fischer in 1891 [10]. At the present time, the Cambridge Structural Database contains a few thousand entries for carbohydrate crystal structures, among which a limited number of molecules are relevant to glycobiology.

With the exception of sucrose and cyclic compounds, such as cyclodextrins or cyclo-amyloses, carbohydrates are reluctant to crystallize in form and size suitable for X-ray crystallographic analysis. This is even more pronounced for compounds having molecular weights ranging from 1000 to 5000 Da. Among the reasons, there is the difficulty to produce sufficient amount of material or the intrinsic occurrence of molecular disorder in solution, where several forms coexists (linear, five and six membered rings, anomeric mixture, etc.). It is also true that much less effort has been devoted to the production of organic crystals of medium sized biomolecules compared to biological macromolecules. Nevertheless, in many instances, ordered samples may be obtained, either in the form of molecular crystals of micrometric dimensions or in the form of polycrystalline materials.

### Small molecule crystals

In the quest to solve the crystal structures of cello-oligosaccharides, as model compounds of cellulose, several attempts to grow crystals of β-D-cellotetraose of a size suitable for X-ray diffraction had failed. Despite many attempts, the best crystals ever obtained for cellotriose and cellotetraose were very thin laths having dimensions of only about 10 µm in thickness. In the case of cellotetraose, single crystals as small as 0.40 × 0.15 × 0.015 mm could be processed by an X-ray synchrotron beam and 3800 independent reflections were collected. The molecular and crystal structure was solved using molecular replacement methods, and refined to an R factor of 0.048 [11]. Those results were useful in the elucidation of the crystalline structure of cellulose.

**Figure 4:**
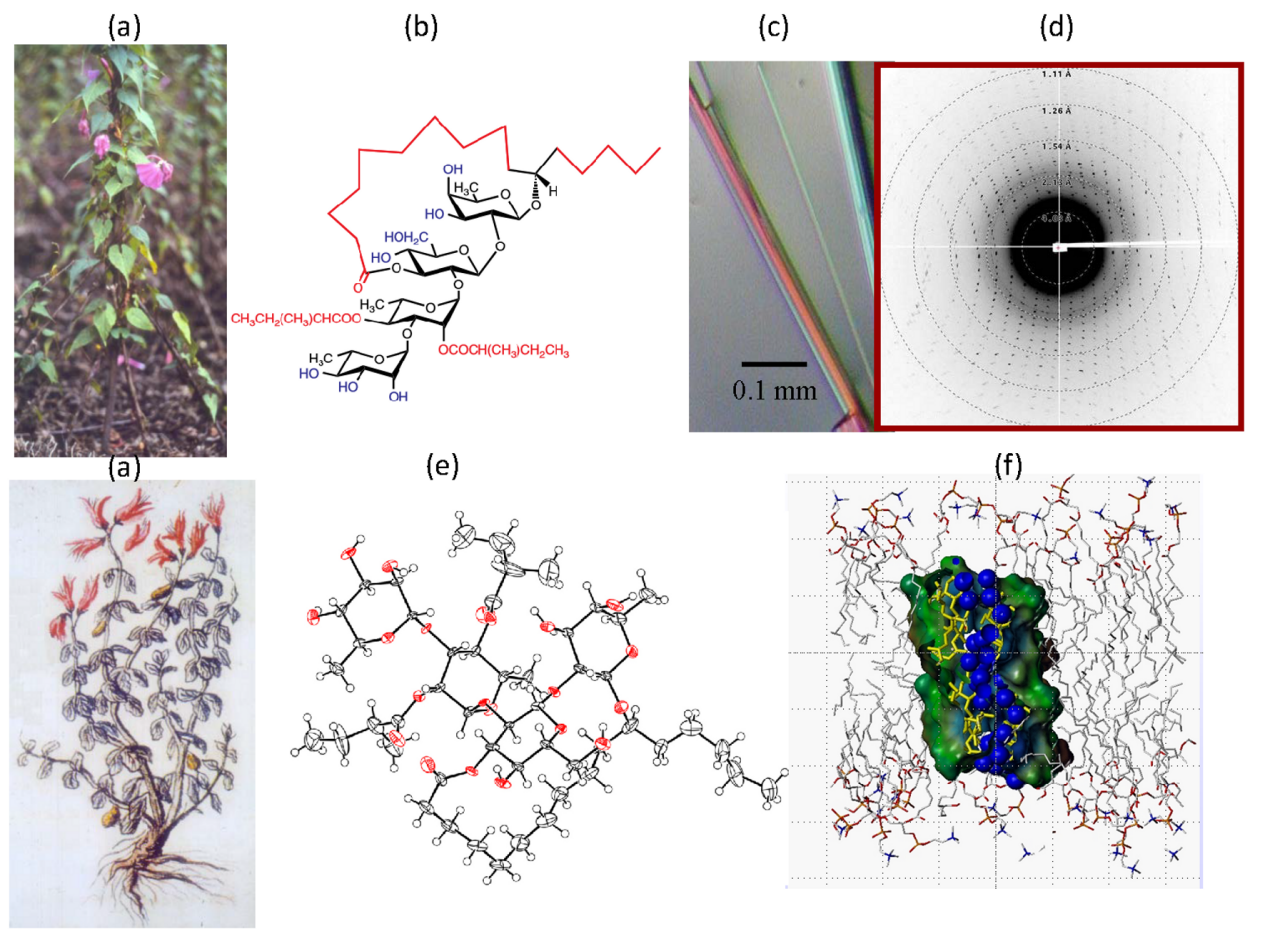
Structural features of the resin glycoside tricolorin A. (a) Extracted from the Mexican variety of the morning glory plant *Impomea tricolor Cav*. (b) Chemical structure of tricolorin A (L-rhamnopyranosyl (1->3)-α-L-rhamnopyranosyl-(1->2)-β-D-glucopyranosyl-(1->2)-β-D-fucopyranoside linked to japinoli acid. (c) Single crystals of tricolorin A. (d) Diffractogram from X-ray synchrotron. (e) Molecular structure of one, out of the four crystallographically independent molecules in the unit cell. (f) Molecular modelling of the insertion of tricolorin A within a fluid phospholipid bilayer [12].

The family of resin glycosides offers another example of difficulty in terms of single crystal growth. Glycolipids (or lipo-oligosaccharides) comprise a carbohydrate moiety covalently linked to a lipid that confers on them an amphiphilic character, which makes them reluctant to crystallize. One member of the family is tricolorin A (L-rhamnopyranosyl (1->3) α-L-rhamnopyranosyl (1->2) β-D-glucopyransyl (1->2) β-D-fucopyranoid linked to japinoli acid forming a 19-membered ring macrocyclic ester, extracted from *Convolvulaceous* species which have been used in traditional medicine throughout the world since ancient times. Small crystals, with dimensions of 0.5 × 0.01 × 0.01 mm, could be grown using protein crystallization methods. Data were collected using synchrotron radiation, and the structure was solved using direct methods. Four independent molecules were found in each asymmetric unit (which contains 284 non-hydrogen atoms) in a highly hydrated unit cell (Figure 4) [12].

### Polycrystalline material

Powder diffraction is a standard technique in material science that is used to investigate polycrystalline materials as many micrometer-sized crystals instead of a large single crystal. A powder diffraction pattern captures all possible crystal orientations simultaneously. The development of synchrotron radiation instrumentation dedicated to powder diffraction [13] allows to perform the experiments that were considered to be impractical before.

**Figure 5:**
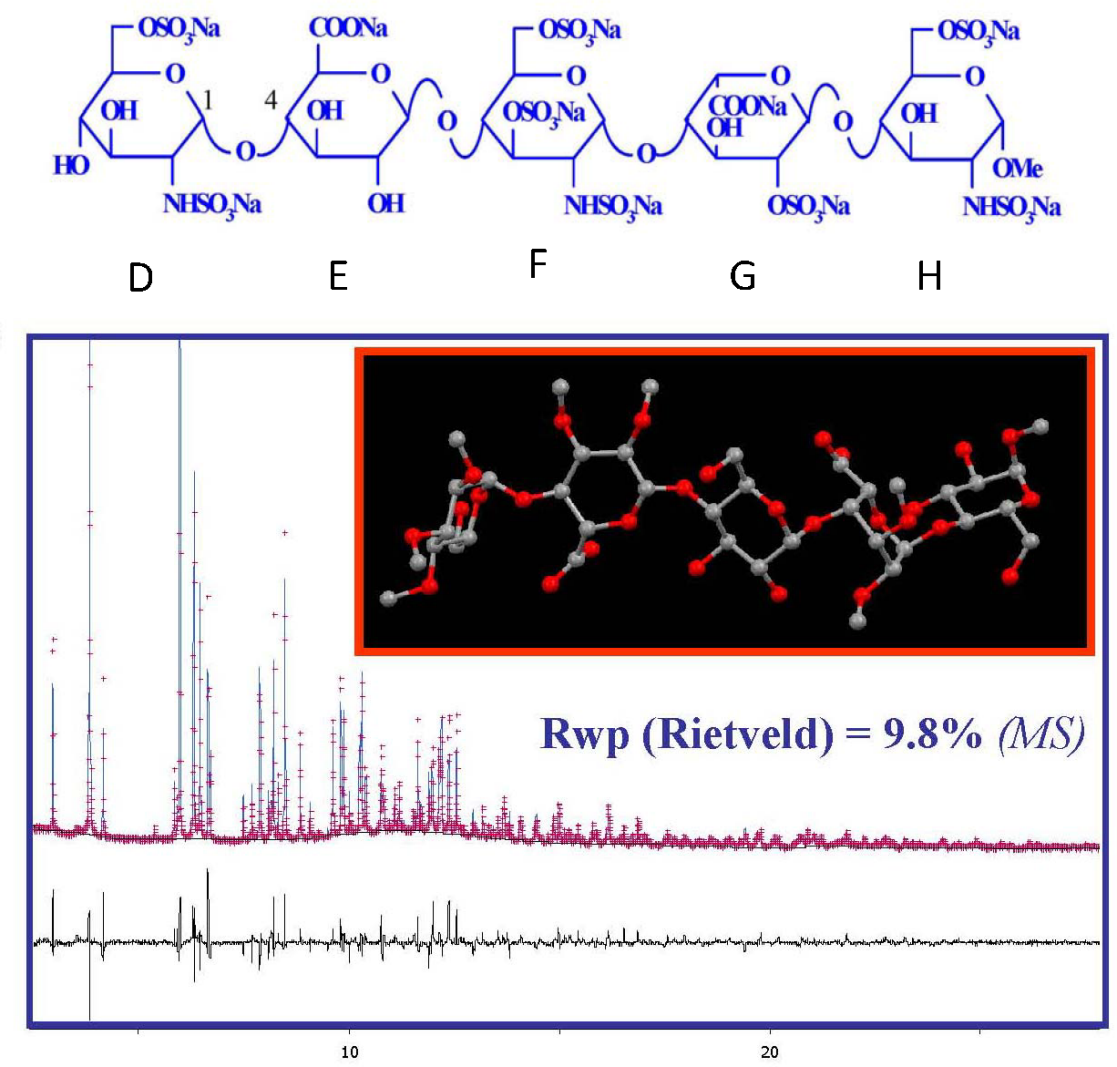
Powder diffractogram measured on a synthetic pentasaccharide from heparin, at ESRF beamline ID31, λ: 0.8 Å). The unit cell constant and the space group symmetry were assigned to: *a* = 15.54; *b* = 8.83, *c* = 17.67, β = 94.6; Monoclinic, *P*2_1_. A 3-dimensional model of the structure in the unit cell was obtained using a molecular model where the sulfated idose residue was kept in ^1^C_4_ conformation [14]. (Courtesy Drs J. Kieffer and Philippe Ochsenbein with permission).

When using high quality data in conjunction with advanced computational methods, it is possible to solve and refine crystals structures of small organic molecules with limited torsional freedom. This approach is less powerful than single crystal diffraction because of a loss of information by reducing the 3D space on a 1D spectra. Nevertheless, the resolution of the crystalline structure of a synthetic pentasaccharide from heparin, illustrates the potential of this technique. From the experimentally recorded X-ray powder diffractogram (Figure 5), the unit cell dimensions and the space group were determined. The process was continued with a computational building of the pentasaccharide and a simulated annealing procedure in direct space to locate the molecule in the unit cell. Once the carbohydrate backbone was positioned, the refinement continued by an adjustment of the rotations of the glycosidic linkages and side chains. The final construction and model completion provided the crystal and molecular structure with a high confidence factor [14].

Recently remarkable examples of protein structures determined by using this technique were also reported [15–17]. The clear advantage over single crystal diffraction is the easier preparation of the crystalline samples. As a result of improvement in the technique’s high resolution, new possibilities exist, such as the investigations of the occurrence of phase transitions in large macromolecules as a function of temperature.

### Macromolecular structures

X-ray diffraction with synchrotron radiation is the most powerful method for revealing the three-dimensional structure of biological macromolecules. Among the 128,000 structures deposited in the Protein Data Bank (January 2017) more than 80% have been measured and solved at synchrotron radiation facilities [18].

Macromolecular crystallography beamlines underwent a constant evolution over last decade that had a dramatic impact on the throughput and on the complexity of the structures determined. However, despite the development in nano-volume liquid handling for high-throughput screens, the crystallization of biological macromolecules still represents an important bottleneck in structure determination. Nanoliter handling devices allow the screening of hundreds of crystallization conditions even with a limited amount of sample of a few tens of microliters [19]. Furthermore, a successful example of automation in crystal harvesting were recently reported [20], while robots are now used to handle cryo-cooled samples at most synchrotron sources. Automation allows for reliable sample exchange and the evaluation of hundreds of samples per day. The development in pixel array detector technology has reduced the time for data collection to minutes or less and significantly improved the quality of the data thanks to no read-out noise and point spread across the pixels. Furthermore the advent of microfocus [21] and microbeam beamlines [22–23] completely dedicated to macromolecular crystallography permits diffraction data collection from smaller samples, of the order of a few micrometers. By matching the X-ray beam to the crystal size, it maximizes the diffraction signal-to-noise and reduces background scattering from crystal holder and mother liquor. New beamline graphical control software [24–26] facilitates beamline operation without exposing the complexity of the hardware. This allows the implementation of elaborate experiments even to users that are less familiar with computational tools. Beamline control software is interfaced with a laboratory information management system (LIMS), a metadata management system [27]. It is used to track samples, record experiment details and report experimental protocols and results from automatic post-experiment data processing protocols [28]. The synergy among these components has recently given rise to completely automated data collection experiments [29].

### Glycoproteins

In recent years, the expression and production of recombinant proteins was of great benefit to the whole structural biology community, with more than 85% of the protein structures deposited in the Protein Data Bank being expressed in *Escherichia coli*. However, many proteins require post-translational modifications for correct biological activity and it is estimated that more than 50% of all human proteins are glycosylated, whereas proteins expressed in *E. coli* do not contain any glycan chains. For proteins that require post-translational modification, eukaryotic expression systems are usually preferred [30].

The crystallization of glycoproteins faces several obstacles, including the micro-heterogenity of glycans at the surface of the protein. For a given glycoprotein, there may exist considerable variations of N-linked glycan chains from protein to protein. Such a heterogeneous macromolecular mix is not suitable for crystal formation. Large post-translational modifications also have the effect of increasing surface entropy and hinder crystal packing. For this reason, it is sometime necessary to manipulate the glycoform to facilitate the crystallization. In the case of the human IgE-FcεRIα [31], Man5-GlcNAc-GlcNAc-Asp-linked glycoforms produced better crystals than in the case where only the Man-GlcNAc-GlcNAc-Asp form was present. There are other cases where crystallization may be facilitated by the presence of glycans that form stabilizing intermolecular contacts within the crystal. Platforms for the expression and crystallization of glycoproteins are available and can typically be successful in a few weeks [32].

**Figure 6:**
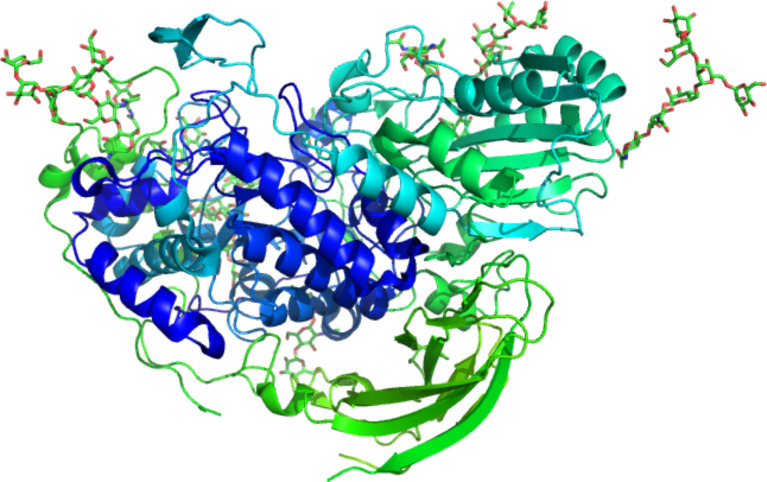
Three dimensional ribbon representation of a heavily N-glycosylated *Aspergilllus* sp. Family GH3 β-D-glucosidase protein (PDB 5FJI) [33].

Nevertheless, in the large majority of glycosylated structures, only the electron density map of the initial N-linked GlcNAc is present and can be modelled. In most of the cases, the glycan chains are exposed to the solvent and highly flexible. In such instances, the glycan can be modelled only up to the last visible residue (Figure 6).

The rise in the deposition of glycosylated protein structures reinforced the need for appropriate model restraints for model building and refinement crystallographic software. Model refinement without correct restraints will nearly always result in distortion and particular caution should be given to crystallographic reports where there is a wrong linkage distance specification or a mistaken anomer and handedness. Automated detection, building and validation of sugar models starting from X-ray diffraction data are being implemented [34].

### Carbohydrate interacting proteins

**Figure 7:**
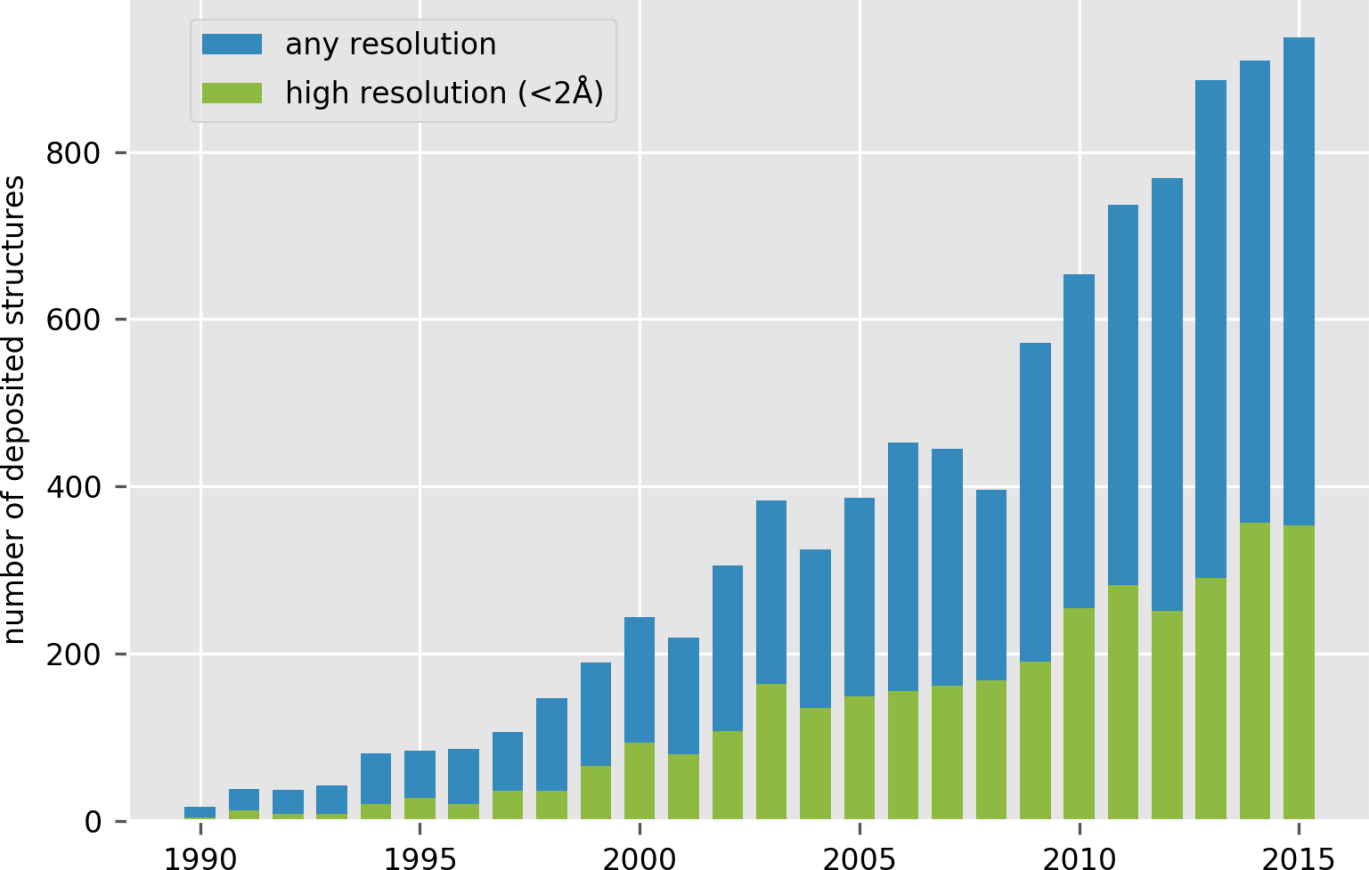
Histogram of the number of deposited crystal structures of glycan-binding proteins deposited over the years. Structures being resolved at high resolution (<2 Å) are displayed in green. (Courtesy Dr. J. Hendrickx with permission).

The carbohydrate-mediated recognition events that have a high biological relevance give a pivotal role to the study of protein–carbohydrate interactions. Those interactions drive several distinct biological events, going from the enzymes involved in the biosynthesis, to the hydrolysis and modifications. Transporters and proteins purely involved in recognition (lectin, antibodies, carbohydrate binding modules, glycosaminoglycan binding proteins) are the other important classes of carbohydrate-binding proteins. Figure 7 shows the evolution of the number of carbohydrate interacting proteins that have been solved over the last 25 years, with a particular emphasis on the number of structures determined at high resolution.

**Figure 8:**
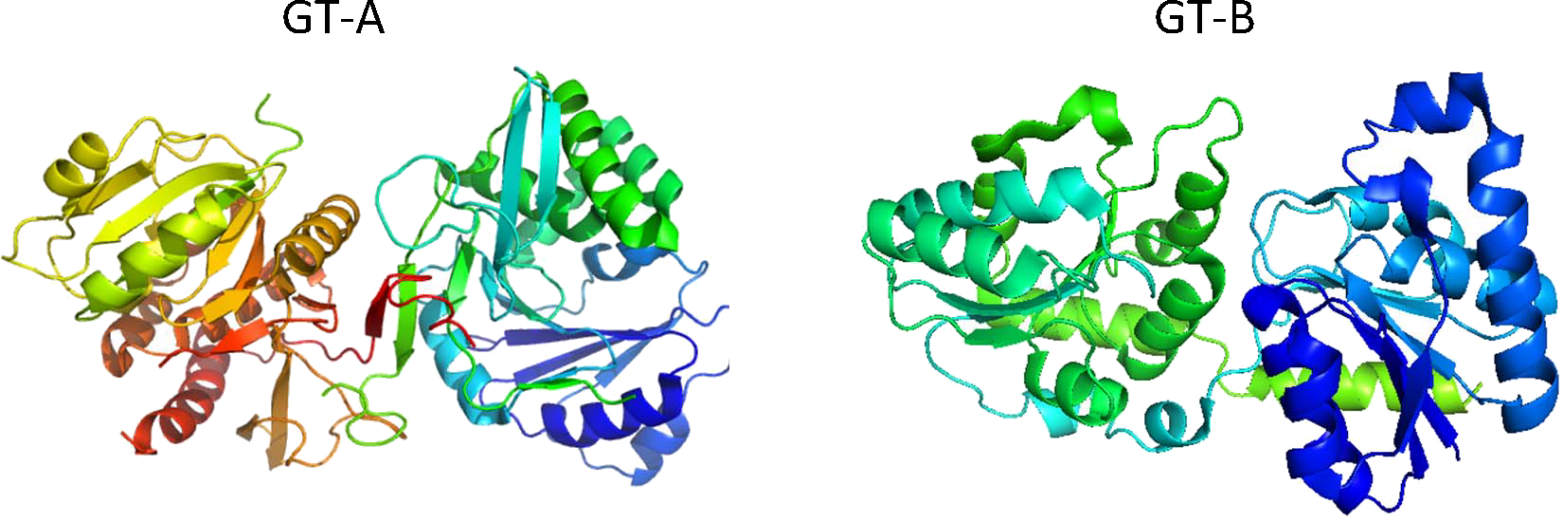
Ribbon diagram representations of prototypical members of the GT-A and GT-B super-family fold, respectively. PDB 1OMZ [38] and PDB 1NLM [39].

**Glycosyl transferases:** The biosynthesis of oligosaccharides is performed by a ubiquitous class of enzymes: the glycosyl transferases (GTs). The catalytic mechanism underlying the biosynthesis of glycosidic linkage requires the transfer of a sugar residue from a donor to an acceptor [35]. Acceptor substrates are carbohydrates, proteins, lipids, DNA, flavonol, antibiotics and steroids. In contrast, glycosyl donor substrates are mostly sugar nucleotides, such as UDP-GlcNAc, UDP-Gal, GDP-Man, and the GTs that process them are often referred to as Leloir enzymes. In certain cases, lipid-linked sugars, e.g., dolichol phosphate saccharides and unsubstituted phosphates are also utilized. The transfer of saccharides by GTs is regio-specific and stereo-specific: depending on the anomeric configuration of the transferred saccharide, two possible stereo-chemical outcomes occur, either inversion or retention. Based on the CAZy classification, the number of GT families amounts to 90, in a context where sequence homology is low http://www.cazy.org) [36]. The increased number of sequenced genomes is paralleled by an increasing number of accession entries for the GTs crystal structures in the PDB, which amounts to 900. Unlike glycoside hydrolases which display a large variety of different folds, the structures of GTs solved today can be clustered in two types of folds (and variants of these folds), namely GT-A and GT-B (Figure 8). Different folds are nevertheless observed for GTs that use lipid phosphate donor substrates. The achievement of the enzyme-transition state complex requires a particular arrangement of the active site that is the result of concomitant protein dynamics, plasticity of GTs and conformation changes that allow for substrate recognition and catalysis [37].

**Figure 9:**
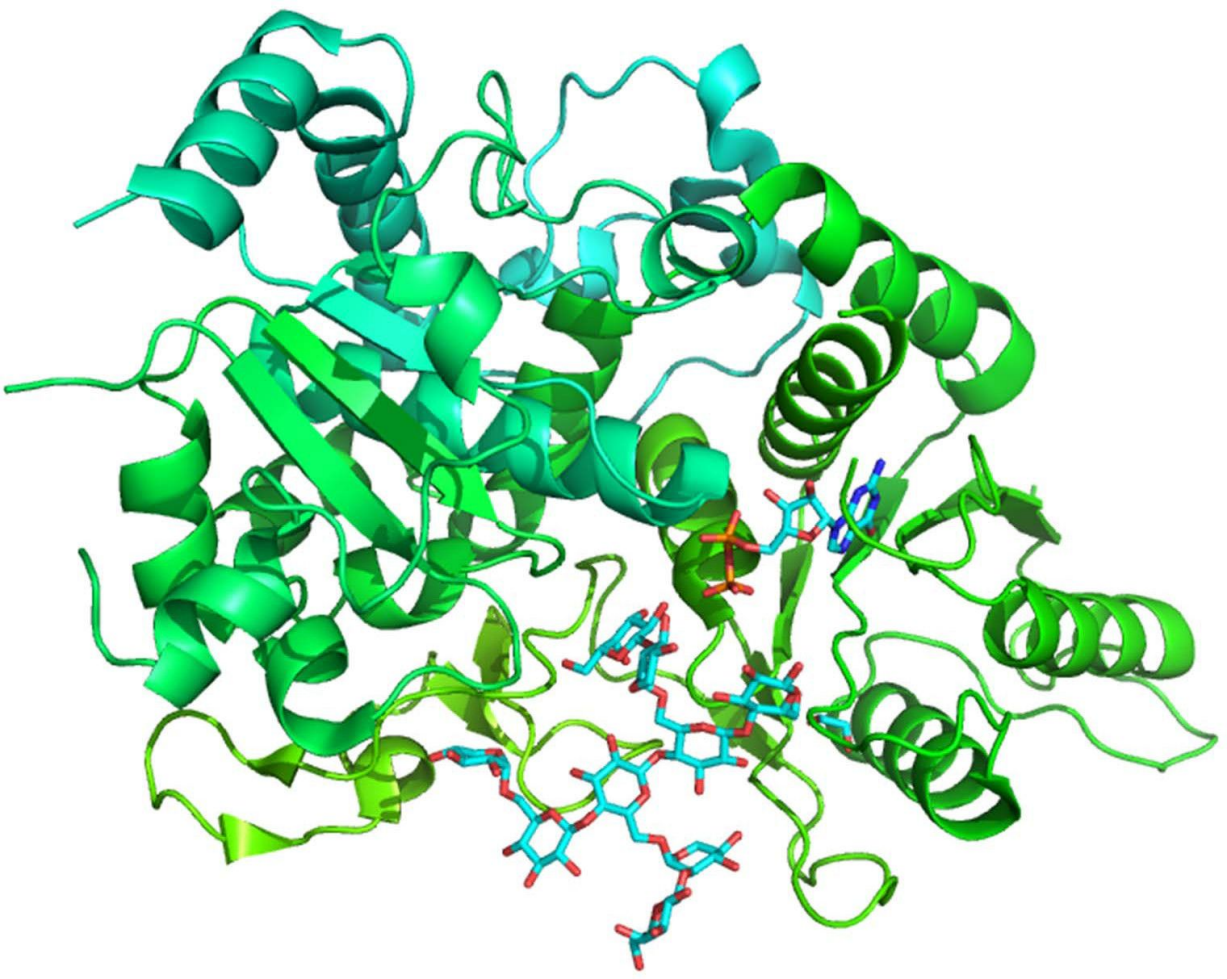
Representation of the FUT1 structure determined in complex with the acceptor (carbon atoms in green) and with end product GDP (carbon atoms in yellow) (PDB 5KOR) [40].

In plants, GTs are also involved in the biosynthesis of hemicellulose. Xyloglucan is one of the main hemicellulose components in the cell walls of dicots. Its biosynthesis involves different GTs, including a fucosyltransferase, FUT1 that belongs to the glycosyltransferase family 37. The determination of the crystal structure revealed yet another variant of a GT-B fold and could explain FUT1 substrate specificity (Figure 9). Furthermore, the determination in complex with a minimal xyloglucan oligosaccharide acceptor and GDP lead to the understanding of the FUT1 mechanism [40].

**Carbohydrate esterases:** Carbohydrate esterases perform the de-O or de-N-acylation of carbohydrates. From a mechanistic point of view, this family of enzymes is divided into two classes, according to the dual role played by the carbohydrate. One class is exemplified by the pectin methyl esterase in which case the carbohydrate plays the role of the “acid”. In another class, the carbohydrate acts as an alcohol, as in acetylated xylan. A classification based on amino acid sequence similarities has been proposed yielding 16 families [41]. Among the 100 crystal structures which have been solved, 30 were obtained in complex with carbohydrates, mainly pectic oligosaccharides.

**Polysaccharide lyases:** Polysaccharide lyases (PLs) constitute a family of enzymes that cleave uronic acid-containing polysaccharide chains. The underlying mechanism is a β-elimination mechanism which generates an unsaturated hexenuronic acid residue and a new reducing end of the polysaccharide. At the present time, the reported number of crystal structures amounts to 190, among which 64 are complexed with carbohydrate ligands. These enzymes show a large variety of folds. Based on amino acid sequence similarities, polysaccharide lyases have been classified in 24 families [41].

**Figure 10:**
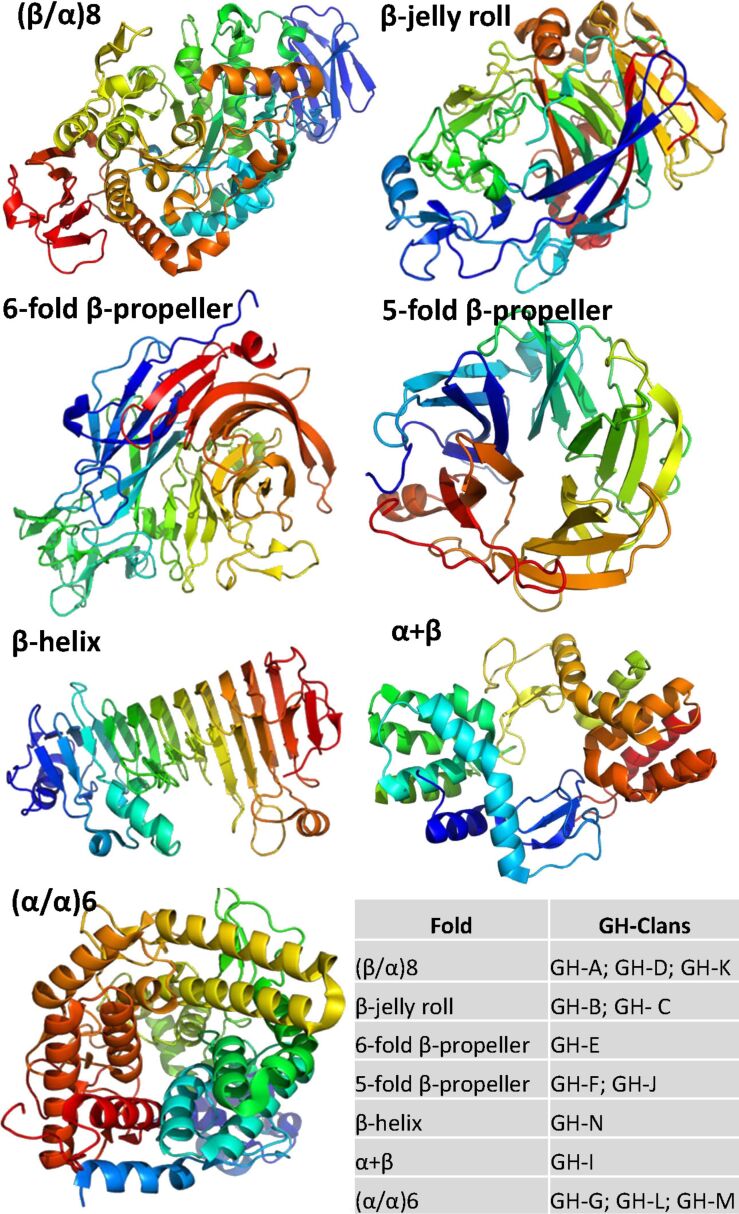
Representation of the seven folds most commonly found in glycoside hydrolases. From the classification of glycoside hydrolases into more than 100 families, a hierarchical clustering into 14 clans has been proposed based on similarities of folds [42]. Some folds are common to different clans.

**Glycoside hydrolases:** The hydrolysis of carbohydrates is the result of the action of a wide spread group of enzymes: the glycosyl hydrolases (GHs). GHs cleave the glycosidic linkage between two or more carbohydrates or between a carbohydrate and a non-carbohydrate moiety. They can catalyse the hydrolysis of O-, N-, S-linked glycosides, as well. The catalytic event can occur either in the middle (-endo) or at the end (-exo) of the substrate. The hydrolysis of the glycosidic bond implies a general acid (proton donor) and nucleophile/base, and involves two amino acid residues of the enzyme. Depending upon the position of these catalytic residues with respect to the substrate cleavable bond, the outcome of the reaction is either an inversion (inverting mechanism) or a retention (retaining mechanism) of the anomeric configuration. At the present time, about 4500 crystal structures of GHs have been deposited in the PDB. Approximately 30% of them are complexed with carbohydrate ligands. A classification of GH (more than 100 families) has been established, first based on amino acid sequences similarities and further consolidated by the availability of 3D dimensional structures [42]. The analysis of the GH structures present in the CAZy database helped not only to decipher the hydrolytic mechanism, but also reveal the evolutionary relationships between these enzymes. An extended classification based on the fold of the proteins, allowed the identification of 14 main clans (Figure 10).

**Carbohydrate binding modules:** Carbohydrate binding modules (CBM) are defined as a sequence of contiguous amino acids within a carbohydrate-active enzyme with a discrete fold having carbohydrate-binding activity. Initially CBMs were classified as a cellulose-binding domain, but their occurrence in other carbohydrate active enzymes required a dedicated classification, separate from other non-catalytic proteins, and similiar to lectins, antibodies and sugar-transport molecules. Depositions in the PDB for CBMs amount to 900. In the CAZy database, CBMs are classified within 80 families based on amino acid sequence similarities, while a three-dimensional structural classification clusters CBM into seven fold families [43]. The most represented fold is the β-sandwich comprised of two β-sheets, each consisting of three to six anti-parallel β-strands. As a large proportion of crystal structures are complexed with carbohydrates (from monosaccharides to oligosaccharides), three CBM types have been classified based on their sugar recognition modes: surface binders, ”endo-type” binders and “exo-type” binders [44].

**Lectins:** Lectins constitute a unique and diverse family of proteins that reversibly bind monosaccharides and oligosaccharides, with utmost specificity, without displaying any catalytic or immunological activity. At the present time, the number of crystal structures of lectins deposited in the PDB amounts to about 1,500. Interestingly, about 60% of them were obtained ligated to carbohydrates, which range from monosaccharides to 10 residue-long oligosaccharides. Lectins occur in plants, animals, algae, bacteria, fungi and yeasts, and viruses. Their involvement in key biologically-important recognition processes is well documented, as in the case of embryogenesis, fertilization, inflammation and metastasis. Lectins play a key role in parasite-symbiotic recognition in microbes and invertebrates of plants and vertebrates. The present role assigned to lectin lies in their ability to decipher sugar-encoded information, i.e., they are a molecular reader of the glyco-code.

**Figure 11:**
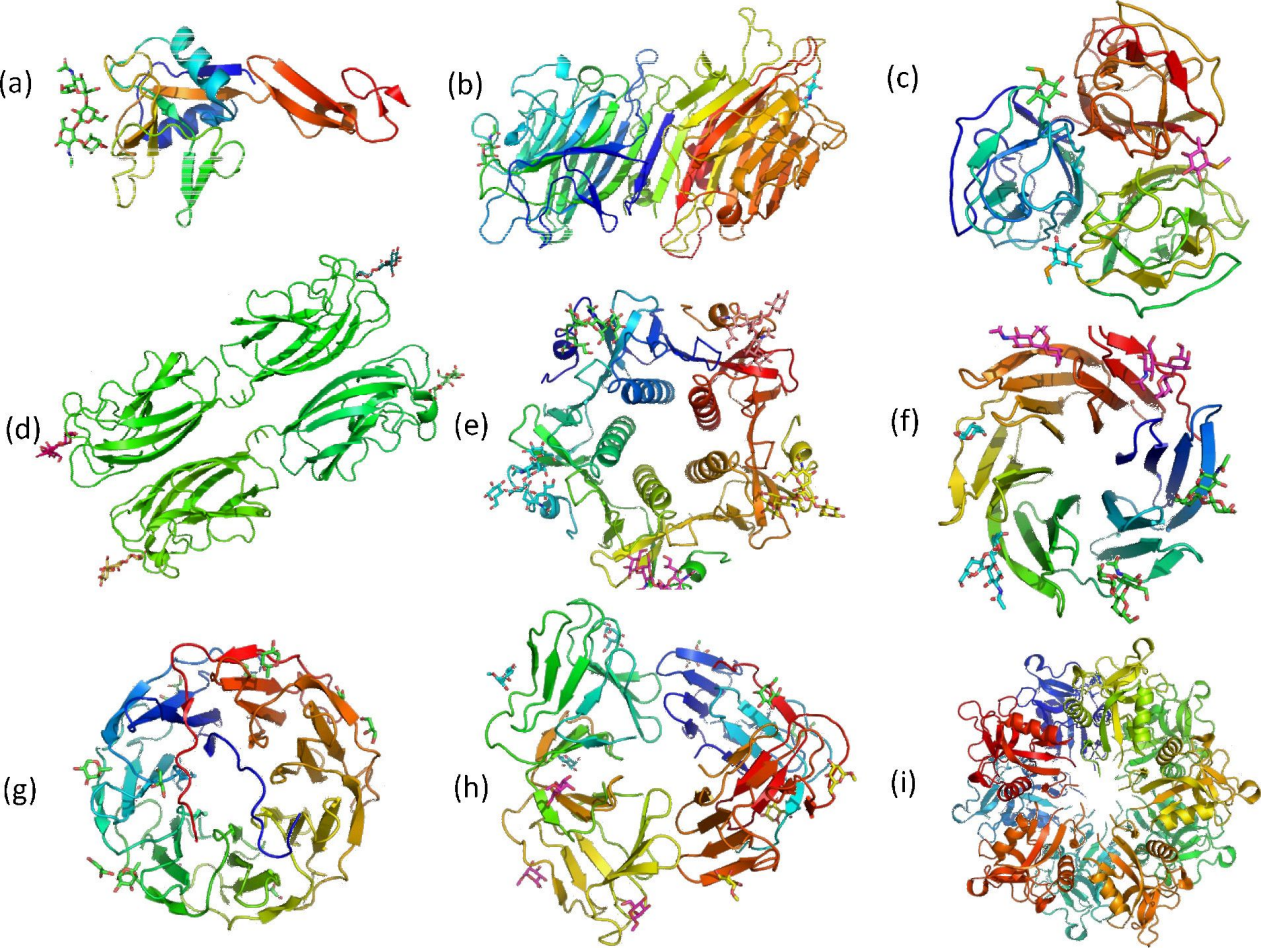
The multivalent carbohydrate binding features of lectins from X-ray structures. (a) Monovalent. E-selectin with bound sialyl LewisX: NeuAc α2->3 Gal β1->4 (Fuc α1->3) GlcNAc (PDB: 1G1T) [46]. (b) Divalent. *Dolichos bifluorus* seed lectin in complex with the blood group A trisaccharide (PDB: 1LU2) [47]. (c) Trivalent. N-terminal domain of BC2L-C lectin from *Burkholderia cenocepacia* with specificity for fucosylated human histo-blood group antigens (PDB: 2WQ4) [48]. (d) Tetravalent. *Pseudomonas aeruginosa* II lectin complexed to iso-globoside Gal α1->3 Gal β1->4 Glc (PDB: 2VXJ) [49]. (e) Pentavalent. Cholera toxin B subunit bound tGM1 pentasacharide: Gal β1->3 GalNAc β1->4 (Neu5Ac α2->3) Gal β1->4 Glc (PDB: 3CHB) [50]. (f) Hexavalent. *Burkholderia Ambifaria* lectin (BambL) complexed with H type2 trisaccharide, Fuc α1->2 Gal β1->4 GlcNAc (PDB: 3ZZV) [23]. (g) Heptavalent. Lectin from *Photorhabdus luminescens* complexed to L-fucose. PDB: 5C9P) [51]. (h) Octavalent. Lectin from *Galanthus nivalis* complexed with Me α-D-Man (PDB: 1MSA) [52]. (i) Decavalent. C-type lectin from *Bothrops jararacussu* (PDB: 5F2Q) [53].

The plethora of three-dimensional structures of lectins, both in unbound form or complexed with oligosaccharides, lead to their organization in a dedicated database, available at http://glyco3d.cermav.cnrs.fr [45]. Information contained in the database provided description of the main features of this important class of proteins. Lectins exhibit a variable oligomeric assembly that ranges from mono- to deca-valency (Figure 11).

Lectins present sugar-binding sites that are in most cases relatively shallow, and are located near the surface and therefore accessible to solvent. One or two calcium ions, identified in several lectin families of different origins, are involved in the carbohydrate binding by direct coordination to the sugar hydroxy groups. The comparison of detailed conformational features of oligosaccharides and their modes of interaction with the protein led to the development of different molecular modelling methods.

A somehow indirect application of the fine specificity of the binding of oligosaccharides to lectins has been elegantly developed to solve the phase problem in protein crystallography. Selenium-labelled carbohydrates can bind to the combining site of lectins at relatively low concentration, and provide sufficient anomalous signals for MAD or SAD methods of phasing to work, as was exemplified by the structure solution of the F17-G fibrial adhesion [54]. This elegant approach was used to elucidate the crystal structure of *Ralstonia solanacearum* lectin [55], *Parkia platycephala* lectin [56] and *Psathyrella velutina* lectin [57].

**Anti-carbohydrate antibodies:** Carbohydrate determinants are expressed on the cell surface through glycoproteins and glycolipids where they are exposed to a wide range of contexts, surroundings and surface densities. It is within such a landscape that antibodies recognize carbohydrate determinants. Data from many systems have shown that the minimum epitopes are often found at the extremity of the determinant. As a result, the presentation of the carbohydrate on the target cell may be such that antibodies with similar specificity exhibit different selective cell-profiling. Up to now, crystallographic studies of carbohydrate-antibodies mainly concentrated on systems where carbohydrates are complexed with antibody (Fab) or variable fragments (Fv). The organization of a small database of high-resolution three-dimensional structures of carbohydrate–antibody complexes [45] provides a way to classify the different types of bindings. Antibodies that recognize a terminal carbohydrate motif present a cavity-like binding feature, while a groove-like binding site is found in antibodies that bind to internal carbohydrate motifs. This is a mechanism typically found in bacterial polysaccharides. There is an occurrence of very large cavities which are open at both ends. The “side-on” entry of the antigen is often at the origin of the occurrence of conformational antigens.

**Glycosaminoglycan–protein complexes:** As members of the proteoglycan family, glycosaminoglycans (GAGs) are linear polysaccharides, constituted by 40 to 100 repeating disaccharide units, which are usually found to be linked to core proteins. These polysaccharides are components of the peri- and extracellular matrix and are present on surfaces or close to surfaces of animal cells. Based on their core repeat disaccharide units, glycosaminoglycans are classified in four groups: the heparin/heparin group; the chondroitin/dermatan sulfate group, the keratin sulfate group, and the hyaluronic group. With the exception of the later, many sources of structural micro-heterogeneities occur as the epimerization at the C-5 position of uronic acids, and N- and O-sulfation. Of paramount interest is the elucidation of the role of GAGs in their interactions with such important proteins as extracellular matrix proteins, chemokines, growth factors, complement proteins, enzymes, and viruses [58].

**Figure 12:**
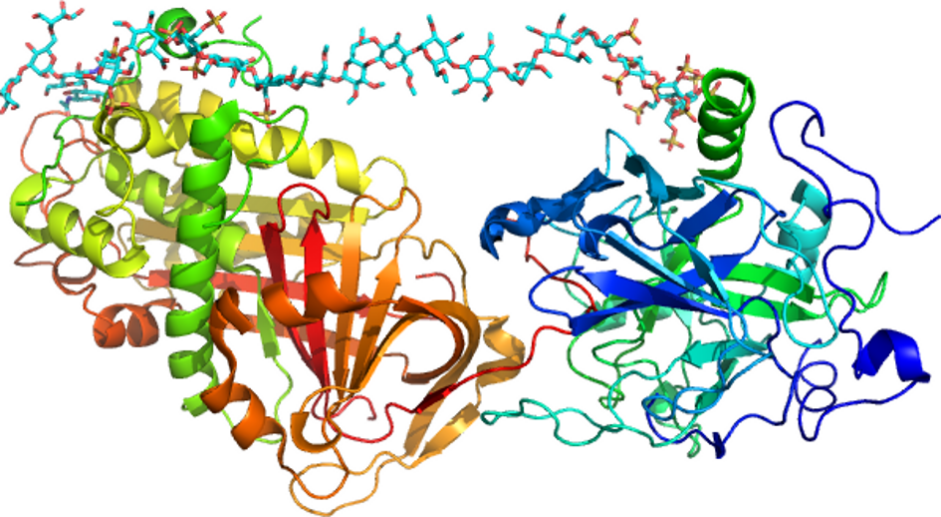
Three-dimensional depiction of the ternary complex formed by a heparin mimetic in interaction with antithrombin. The structure has been solved at 2.6 Å resolution (PDB 1TB6) [59]. The basis of the antithrombotic properties of therapeutic heparin could partly be deciphered by the availability of such a three-dimensional structure.

The three-dimensional structures of proteins co-crystallized in interaction with GAG fragments have been organized in a database (http://glyco3d.cermav.cnrs.fr/). Because of their relevance for pharmaceutical application, most of these fragments are heparin oligosaccharides. Crystallization of proteins in complex with GAGs is very difficult because of the high degree of heterogeneity and intrinsic flexibility of GAGs. The crystal structure of a fragment as long as a hexadecasaccharide could be co-crystallized as complexed with thrombin and antithrombin at 2.5 Å resolution. For the time being, this is one of the largest oligosaccharide structures ever established throughout macromolecular X-ray crystallography (Figure 12) [59]*.*

**Figure 13:**
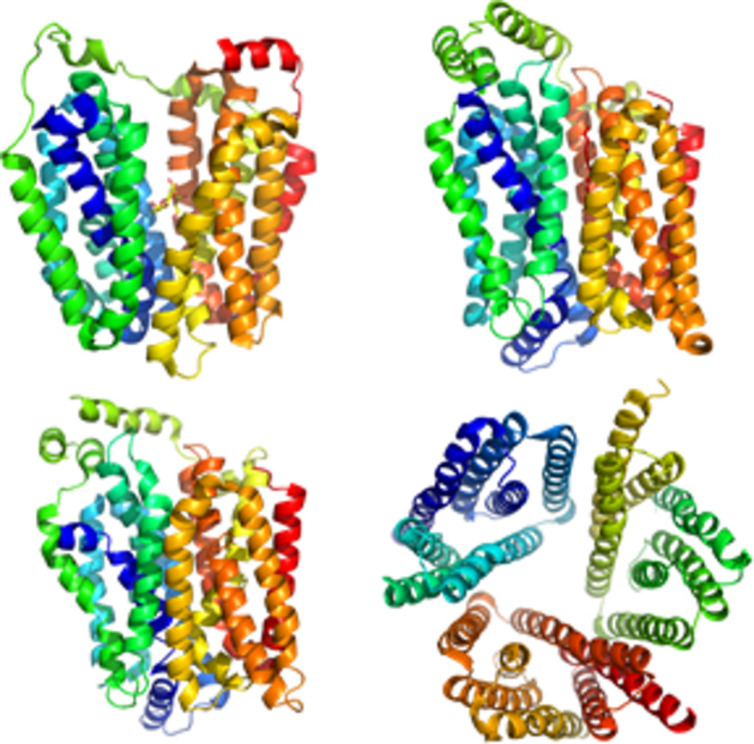
3D representation of different sugar transporter structures: (left to right, top to down) lactose permease structure (PDB 1PV7, [60]), of the human glucose transporter GLUT1 (PDB 4PYP, [61]), of the bovine fructose transporter (GLUT 5) (PDB 4YB9, [73]) and of a SWEET transporter of *Orzyva sativa* (PDB 5CTG, [72]).

**Transporters:** Soluble sugars serve many purposes in complex organisms. Their cellular exchange relies on transport proteins that are responsible for uptake or release. To date, three main families of eukaryotic transporters have been identified GLUTs, SGLTs, and SWEETs – the most recently discovered sugar transport family, which is responsible for cellular export. In mammals, 14 monosaccharide transport proteins GLUTs are responsible for the diffusion of glucose, galactose, fructose, urate, myoinositol, and dehydroascorbic acid. SGLTs are sodium-glucose symporters that couple the transport of glucose to sodium ions. SWEETs have been characterized the most recently. Major carbohydrate transporters mediate an active uptake and efflux of various mono- and disaccharides. The low affinity of these proteins for sugars seems to be a characteristic feature of transporters involved in high turnover rates, rather than a highly specific transport at low levels of substrates. The structure of the first transporter to be determined was the one of lactose permease LacY [60]. Later on, the structures of different bacterial homologues were also solved. It is only recently that the structure of human GLUT1 was reported [61]. Nevertheless, the joint difficulty to solubilize and crystallize membrane proteins, explains the paucity of crystal structures deposited in the database [62–72]. This is even worse for those proteins involved in the transport of sugars (Figure 13)

### Kinetic crystallography

Since the biological activity of many proteins is preserved in the crystalline state, the possibility to investigate the dynamic process of their mechanisms is absolutely intriguing. In kinetic crystallography, a biological reaction is initiated in the crystal and the fates of the transient species formed are followed by determining the structural changes. Depending upon the time scale of the reaction and the scheme used for its initiation, time-resolved crystallography requires either the use of fast diffraction techniques such as Laue diffraction (polychromatic beam), or the capture of intermediates by trapping methods. These trapping strategies require the complementary use of UV–visible single-crystal spectroscopy. Providing extreme care is taken to avoid artefacts, these methods are in principle available to a wide range of biological systems. Two types of intermediate trapping schemes are available.

In the so-called “trigger-freeze” experiment, a large fraction of molecules is brought into the intermediate state of interest at room temperature, which is trapped by flash-cooling. While in a “freeze-trigger” experiment, the sample is first flash-cooled, and then the reaction is triggered potentially after a transient and controlled temperature increase [74].

**Figure 14:**
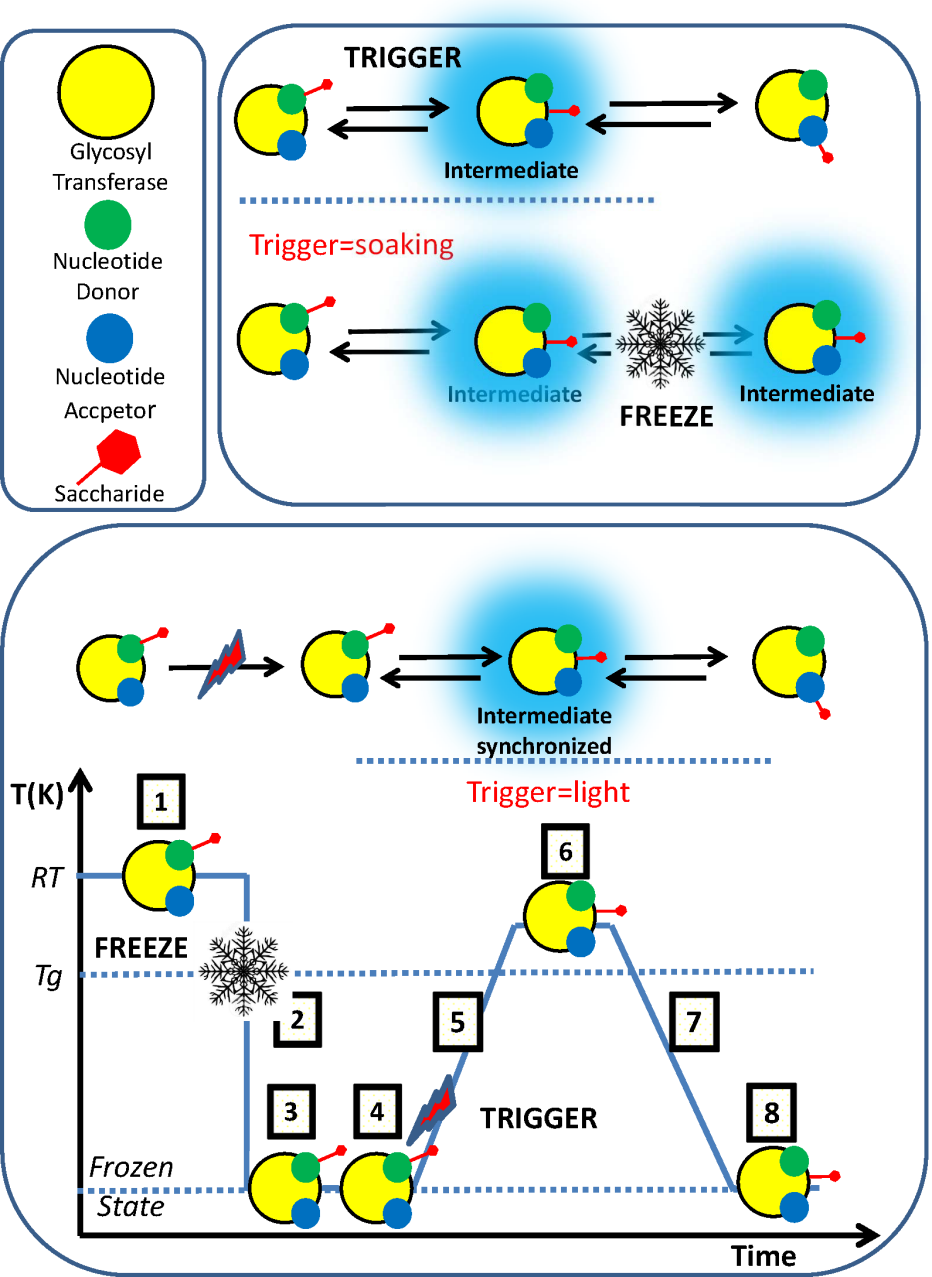
Kinetic crystallography. Protein crystals are soaked with the cage compound (Step 1) followed by flash-cooling (Step 2). The structure determination of the complex is solved (Step 3) to see if and how the cage-compound binds to the protein active site. Step 4 is the cleaving of the cage compound using a laser of appropriate wavelength. In this state, the substrate is available for hydrolysis but the frozen state prevents this from happening. Step 5 consists of a slow increase of the temperature to cross the glass transition and reach a temperature region where the protein has a greater degree of conformational flexibility but with a reaction rate slower than at room temperature. In Step 6, with the enzyme at room temperature, the reaction can proceed with a reorganization of the active site to transfer the sugar to a nucleophilic amino acid. An intermediate is formed and trapped during Step 7, which consists of decreasing the temperature to go back to the frozen state. Step 8 is the structural determination of the protein and elucidation of the intermediate. (Adapted from [75] with permission from Dr. G. Batot).

The ‘trigger-freeze” approach consists in the use of various soaking times for the crystal with substrate sugars in presence of H_2_O or glycerol (“trigger step”) followed by the freezing step ranging from a few minutes to several hours. The use of a “freeze-trigger” approach solves the synchronization issue but introduces experimental complications as a photo-activable analogue called a caged-compound is required. For this, the substrates have to be modified by adding a photolabile group that prevents the reaction from occurring. The principles of the ideal cage-compound based kinetic crystallography experiment are presented in Figure 14.

The studies of the mechanism of blood group glycosyl transferase have been investigated by kinetic crystallography approaches with the aim of characterizing the double-displacement mechanism which involves the formation of a covalently bound glycosyl-enzyme intermediate, by trapping and solving the X-ray structure of this intermediate [75]. The A and B antigenic determinants are synthesised by the blood group A (GTA) and the blood group B (GTB) glycosyltransferases which transfer GalNAc from UDP-GalNAc for the A type and a Gal residue from UDP-Gal for the B-type. A mutant of the galactosyl transferase was created with the capacity to act as GTA and GTB [76–77]. The first attempt was conducted using the “trigger-freeze” method. The experiments were inconclusive presumably due to the lack of synchronisation of the reaction within the crystals and because the reaction time scale is shorter than the time scale of substrate diffusion in the crystals. For example, when UDP-GalNAc was soaked for 24 minutes, experiments resulted in structures with UDP-GalNAc in several conformations that are difficult to interpret. The “freeze-trigger” route was started using a series of cage compounds that had been synthesized. They all included a substrate donor, either UDP-Gal or UDP-GalNAc with an additional group on the sugar or on the uracil. Photolysis at 100 K was monitored by UV–vis absorption, both in solution and in crystals in order to assess the efficiency of the laser ablation in the crystalline glycosyl transferase. The four steps of the “freeze-trigger” process could be validated throughout by elucidation of the crystal structure of the glycosyl transferase, which has the active site occupied in a semi-closed conformation of the substrate with various levels of ordering of the internal flexible loop.

### Small angle X-ray scattering

Small angle X-ray scattering is a universal technique whereby X-rays are recorded that have been elastically scattered at a low angle from samples in solution. Analysis of the scattered X-rays allows low-resolution structural information to be obtained, such as average particle size, distribution and shape. Different kinds of samples beside soluble proteins can be studied by this technique including nucleic acids, protein-based complexes, lipids, membrane proteins and surfactants, glycoproteins, virus, polymers and colloids [78–79].

**Proteins:** SAXS applied to biological materials (BioSAXS) is a complementary tool to protein crystallography and has become an invaluable resource for structural biologists [80]. Although at a much lower resolution than protein crystallography, BioSAXS permits the structural analysis of macromolecules at more physiological conditions, besides being suitable for the study of heterogenous systems that are unlikely to crystallise. Furthermore, the experiments in solution allow the effect of other factors, such as pH, ion concentration, or temperature, on the overall protein structure to be studied. Samples for structure analysis should be highly monodisperse. Besides sample quality control by using complementary analysis, such as dynamic light scattering, native gel, ultracentrifugation, many BioSAXS beamlines at synchrotrons are nowadays equipped with size exclusion chromatography devices directly connected with the sample cell and the data acquisition systems [81].

**Figure 15:**
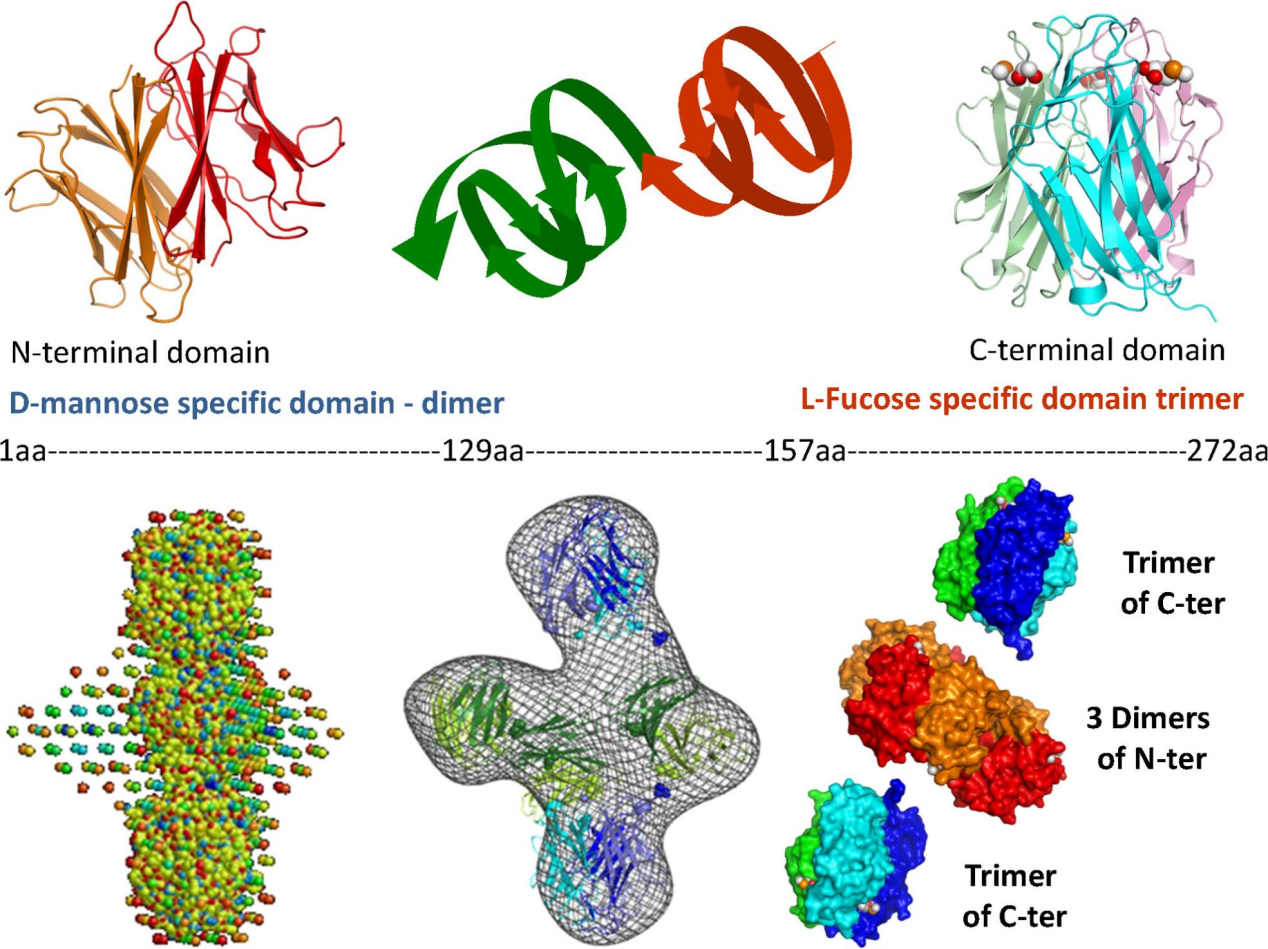
Reconstruction of the full three-dimensional structure of the soluble lectin (BC2L-C) from the opportunistic pathogen *Burkholderia cenocepacia* using Bio-SAXS experiments, from the knowledge of the respective crystalline structures of the N- and C-domains which had been solved separately because a sequence of 38 amino acids in the native protein was too flexible to allow crystal growth [48].

An illustration of how BioSAXS experiments can help to complete data obtained by protein crystallography is given by the characterization of the full structural assembly of the lectin of *Burkholderia cenocepacia,* an opportunistic bacterial pathogen. Throughout biochemical characterization, the lectin, BC2L-C was shown to be composed of two distinct domains, each displaying unique specificities and biological activities. The protein is a super lectin that binds independently to fucosylated human histo-blood group epitopes and to mannose/heptose glycoconjugates. The N-terminal domain is a fucose-binding lectin having similarity with tumour necrosis factor. The structure of the other domain (C-terminal part) which belongs to the superfamily of calcium-dependent lectins displays specificity for mannose and L-glycero-D-manno-heptose monosaccharides. The two domains are linked by a conformationally flexible sequence of 38 amino acids which was detrimental for crystallization. The respective crystal structures of the N- and C-domains could be solved separately and eventually used to establish the overall structure of the assembly by small-angle X-ray scattering (and further confirmed by electron microscopy). Figure 15 displays the reconstruction of the full macromolecular complex as a flexible arrangement of three mannose/heptose-specific dimers flanked by two fucose-specific TNF-α-like trimers [48]. This study (along with many other examples) highlights the potential of SAXS to decipher the global state of glycoproteins and carbohydrate binding proteins in solution so as to greatly amplify the high resolution 3D structural information derived from macromolecular crystallography of domains of small proteins.

**Colloids:** Scattering methods light, neutron and X-ray have long been the methods of choice to investigate the states of soft-condensed materials, which include solutions and gels. Differences in wavelengths and scatterers can be used for combined measurements yielding supplemental information. Following the instrumental developments of light sources, SAXS has become a common tool for the investigations of the state of materials in solution at the nanoscale. Many studies have been devoted to polysaccharides, for which structural change have been observed in real time. There exists extensive literature on this subject and the role played by polysaccharide association structures in food and in biomedical applications, as hydrogels, triggers the development of novel experiments and tools, such as optical tweezers, while making use of synchrotron radiation. Description of the details of molecular interactions occurring between complex materials such as polysaccharides and muccin is among the many new achievements that yields to the rational design of muco-adhesive polysaccharide-based nano-formulations [77].

The availability of new instrumentation that combines wide and small angle X-ray scattering and high resolution ultra-small angle X-ray scattering in a time-resolved manner is creating an opportunity to investigate the microstructure and non-equilibrium dynamics of soft matter on a length scale from a few angtroms to micrometers and on a timescale descending to the millisecond.

### Grazing incidence X-ray reflectometry

**Glycolipids:** Despite their importance in the constitution and dynamics of plasma membranes, the structural and physicochemical features of gangliosides have been somehow neglected presumably because of the lack of appropriate experimental techniques. X-ray reflectometry is a surface-sensitive analytical technique based on the measure of the intensity of X-ray reflected by a flat surface. Any deviation from surface flatness will result in deviation of the reflected beam which can be analyzed to obtain the density profile of the interface normal to the surface [82].

**Figure 16:**
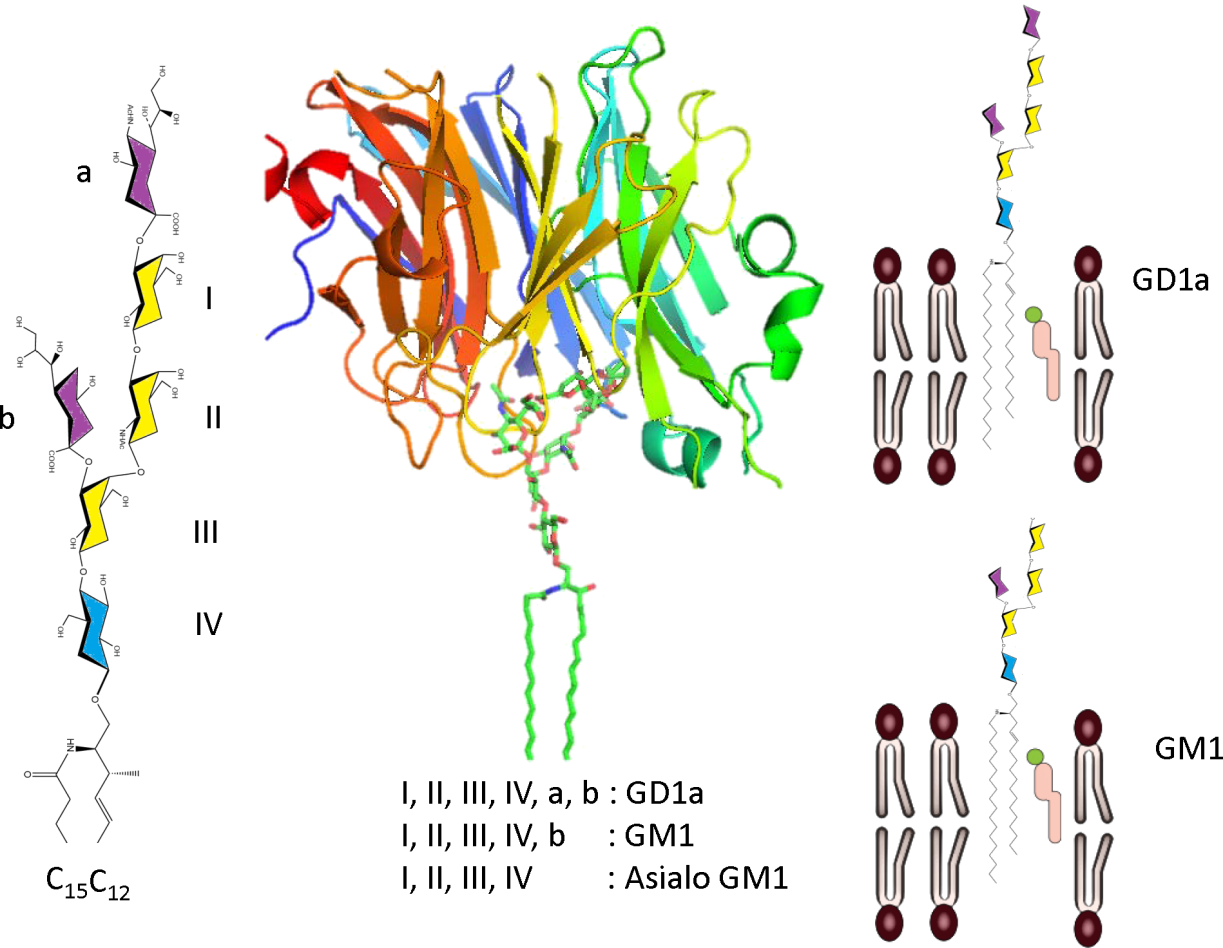
Characterization by synchrotron X-ray reflectometry of the transverse structures of a model membrane incorporating glycolipid rafts, under the action of a sialidase. (Adapted from [83] with permission from Dr. L. Cantù).

Synchrotron X-ray reflectometry has been used to access the transverse structure of a biomimetic plasma membrane incorporating glycolipid rafts. The in situ chemical conversion of GD1a gangloside into its metabolic product under the action of sialidase was investigated. The outcome of the sialidase action is not limited to the creation of GM1 and AsialoGM1 gangliosides as it is accompanied by a reshaping of the membrane which involves a rearrangement of the headgroups on the surface (Figure 16) [83].

### Polysaccharide structures

In contrast to other macromolecules and because of the lack of regular crystalline order, X-ray diffraction of polysaccharides usually leads to an insufficient number of reflections to permit structural determination based on the data alone. Such a lack of experimental data must be complemented by modelling techniques. As such, the process of structural elucidation combines the calculation of diffraction intensities from various low energy models with those intensities collected on X-diffractograms. In this context, it is even most appropriate to use the term ‘model’ in place of ‘structure’. These experimental limitations explain why so few models of polysaccharides have been reported: there are just over 100, counting all polymorphs, variants, derivatives, and complexes. Because of their ubiquitous occurrence, polymorphs of celluloses and starches have attracted most of the attention.

#### Fibrillar structures: cellulose and starch

**Figure 17:**
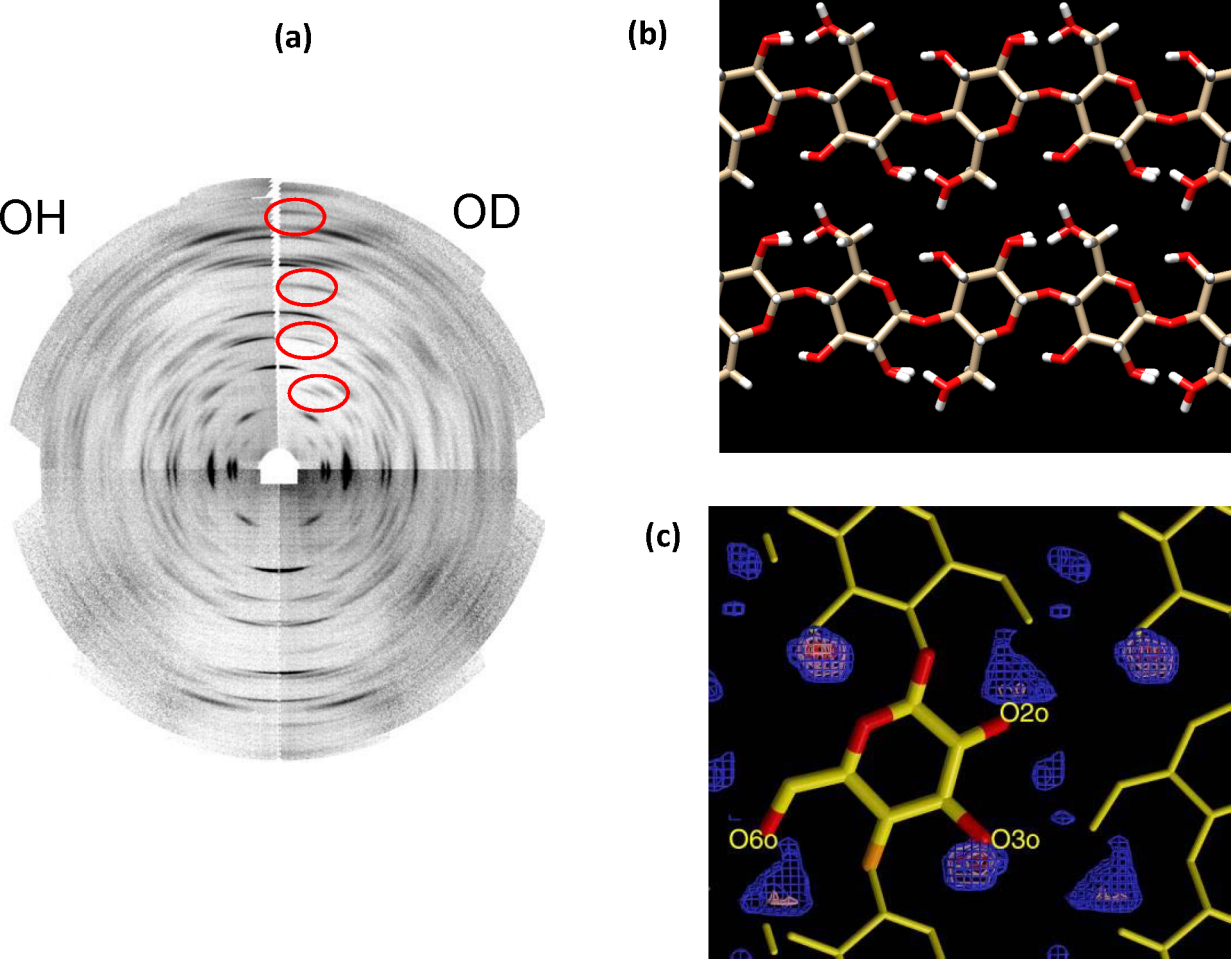
Complementary use of X-ray synchrotron and neutron fiber diffraction to unravel the three-dimensional structural organization of cellulose Iβ from *Halocynthia roretzi* (a). Composite fiber diffractogram of native sample (OH) and deuterated sample (OD). The differences in diffracted intensities are highlighted by the red contours (courtesy of Dr. Y. Nishiyama, with permission). (b) Depiction of conformation of the cellulose chains and their interactions in the unit cell, showing the disordered orientation of primary hydroxy groups. (c) Details of the difference of electron density highlighting the location of the deuterium atoms. Drawn from atomic coordinates taken from reference [85].

**Cellulose:** The first X-ray fiber diffractograms of native cellulose were reported more than one century ago. The results of the investigations that have been undertaken left many of the structural details unclear as conflicting structural models were reported. One particular obstacle to be overcome in the study of cellulose microfibrils is the co-existence of a mixture of two crystal forms Iα [84] and Iβ [85]. In light of this allomorphism, the elucidation of the structure of cellulose I, awaited the mature developments of large scale facilities of synchroton and neutron sources, and the mastering of deuteration methods of the intra-crystalline regions of the native cellulose samples without altering the overall structural integrity. Through an ingenious combination of synchrotron and neutron fiber diffraction, a highly accurate structural model could be established. The samples diffracted to better than 1 Å resolution, and provided the determination of C- and O-atoms positions from the set of X-ray diffracted intensities (Figure 17). In addition, the position of hydrogen atoms were determined from Fourier-difference analysis from the set of neutron diffracted intensities collected from hydrogenated and deuterated samples [85].

This resulted in a description of the three-dimensional features of both allomorphs of native cellulose. Nevertheless, a detailed elucidation of the biosynthetic mechanism is still required to understand the occurrence of two different structural arrangements within the same microfibrils. Some unexpected features still need to be elucidated and this would require the use of complementary methods*.*

**Figure 18:**
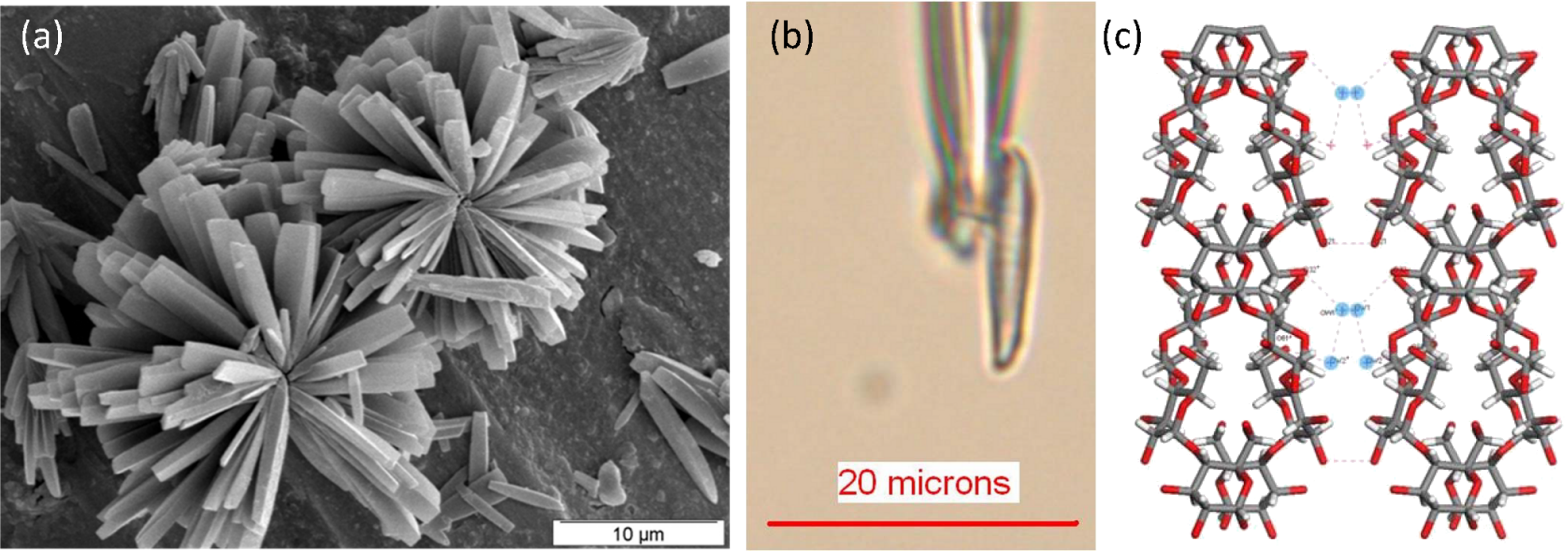
Scanning electron micrograph of high-quality micrometer-sized A-amylose microcrystals grown from short chains of synthetic starch (a) and of a single crystal glued to a borosilicate glass capillary tip (b). (c) Three dimensional representation of the crystal and molecular structure derived from X-ray synchrotron diffraction. (a) and (c) taken from reference [91] with permission from Actualité Chimique (http://www:lactualitechimique.org). (b) (Courtesy, J. L. Putaux; a very similar image of the subject was published in reference [88]).

**Starch:** The complexity of starch in terms of the nature and size of its macromolecular components (amylopectin, amylose) has always been an obstacle to the elucidation of the structural components and their arrangements, which are at the origin of the birefringence of a starch granule. The structure of the crystalline domains of the two allomorphs of starch granules found in cereal and tubers had been established from a series of experimental observations (X-ray and electron crystallography) and molecular modelling. While displaying differences in their mode of interactions, both allomorphs are characterized by a parallel arrangement of parallel-stranded left-handed double helices [86–87]. A second look at the crystal structure of the A-polymorph became possible when microcrystals were grown from short chains of synthetic starch and diffraction data collected using a micron-sized beam at a synchrotron source. While this new investigation corroborated the essential features of the original model, some additional fine details were revealed (Figure 18) [88].

#### Multiscale organization

**Figure 19:**
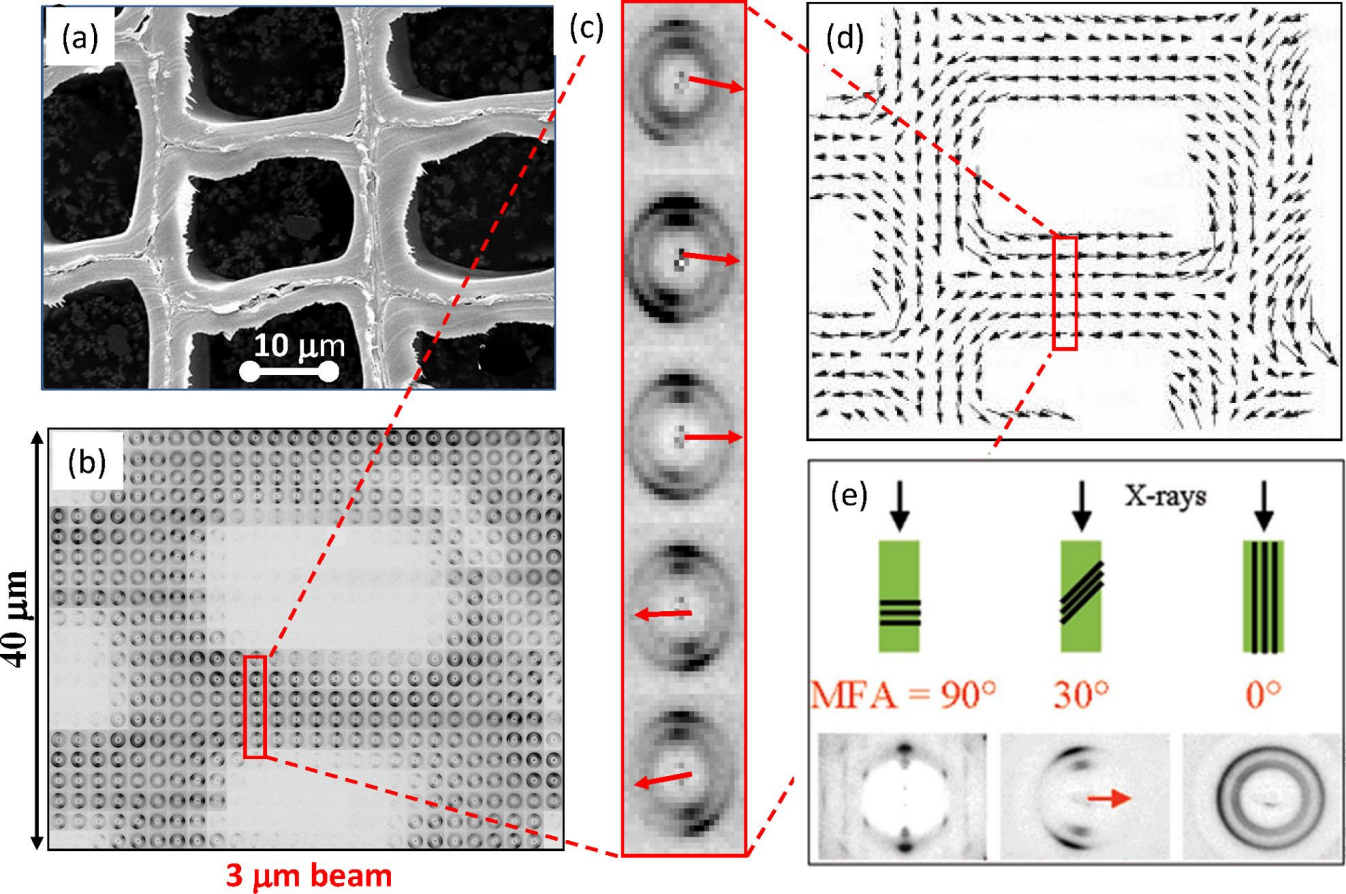
Cartography of distribution and orientation of cellulose in wood using a 3 µm X-ray beam. The scanning of a 10 µm thick wood section, by increments of 2 µm, (a) a collection of “fiber” like diffractograms was collected (b). From each fiber diffractogram, the attribution of the diffraction spots indicates the local orientation of the microfibril axis (c). This is depicted by arrows which indicate a marked local asymmetry in the microfibril (d). The integration of the degrees of disorientation over the full map gives the orientation of the microfibril angle (MFA) along the direction of propagation (e) (adapted from reference [89], and with permission of the International Union of Crystallography, http://journals.iucr.org).

**Cellulose:** Knowledge of the structure of a material is necessary to understand its properties. In the case of cellulose, it is also the key to ascertain the processes of biosynthesis. Cellulosic materials in nature often have many levels of structural complexity, whose organization depends on the source organism. In wood, a cohesive interlaced network of crystalline microfibrils of cellulose composes a first level of interacting components of the cell walls. The typical dimensions of the cellulosic fibers, which are composed of 30–40 cellulose chains, have lengths in the region of 1–2 nm and width of about 35 Å. The elucidation of the structural organization of these microfibrils came from the use of a micro-focused X-ray beam of 3 µm on a wood section of 10 µm thick and oriented perpendicular to the incident beam [89]. As depicted in Figure 19, a complete distribution map of the orientation of the axes of the cellulose microfibril, of a specimen of 52 × 42 µm, was established through a series of 546 diffraction patterns. These results can be translated into three-dimensions and establish the existence of an ultra-structural organisation in which the orientations of the cellulose fibrils follow a super-helicoidal fashion.

TEMPO-mediated oxidation is one of several methods that can be used to extract nascent crystals of cellulose, or cellulose microfibrils from biomass. For this process to be optimal, some fundamental aspects of the structural and ultrastructural characterization of the cellulosic material have to be ascertained. Indeed, the extraction process should be adapted to the specificity of the various sources (e.g., wood, cotton, jute, bamboo, etc.). For dispersions in aqueous suspension, the structure of cellulose nanofibers (and their aggregates) can be characterized by SAXS. This technique has permitted quite significant insight to be gained about the structure of cellulose from a variety of botanical origins. In the case of wood pulp, cellulose nanofibers displayed a ribbon shape of about one micrometer in length. The cross-sections sizes were found to cluster in two groups with dimensions of 3 nm × 8 nm and 9 nm × 20 nm, respectively. Quite different results were obtained for the structure of microfibril fractions extracted from never-dried delignified spruce wood. In this case, the observed morphology was of the type “nanostrips” that had a characteristic thickness and width of about 0.5 nm and 4 nm, respectively. The thickness is an indication that the nanostrips are made up of only one monolayer of cellular material, which indicated the occurrence of “two-dimensional’ crystals that could be further investigated by wide-angle X-ray diffraction [90].

**Starch:** Depending upon their botanical origin, starch granules display an elliptical shape with dimensions ranging from 0.1 to 100 µm. The advent of micro-focus X-ray diffraction from synchrotron radiation offered the possibility to explore the arrangements of the crystalline domains which are at the origin of the birefringence of the starch granule. Using a 2 μm wide X-ray beam, a complete cartography of the relative orientation on a single granule could be drawn. Two-dimensional fiber diffraction patterns were collected for each domain on a grid of 4 × 4 μm. Information about the nature of the crystalline structure (for allomorphs A, B and C) was obtained confirming the orientation of amylopectin double helices in the crystalline lamellae as well as the location of these domains and their relative orientation with respect to the granule. The most detailed investigation performed on potato starch (B allomorph) indicates that the double helices do not seem to point towards a single focus but rather towards the surface of an inner ellipsoid. Thus, the double helices have a radial orientation, and are perpendicular to the surface of the granule.

**Figure 20:**
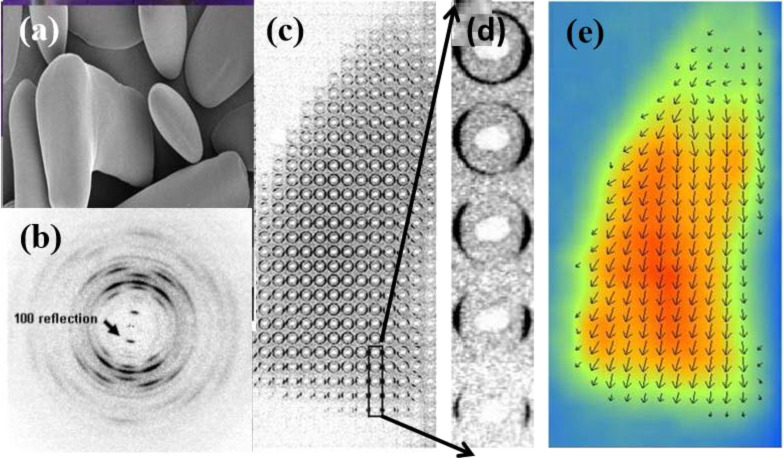
Structural micro-diffraction scanning of a starch granule from *Phajus grandifolius* with dimensions 50 × 200 µm. (a) Scanning electron micrograph. (b) X-ray diffractogram collected from a peripheral region of the starch grain. The width of the azymutal (100) reflection indicates the level of crystallinity. (c,d) The cartography of the crystalline domains collected on a single grain, on a grid having 4 × 4 μm dimensions, using a 1 µm X-ray beam. The total surface explored was greater than 5,000 µm^2^. (e) The width and orientation of the (100) reflection on each diffractogram reflects the level of crystallinity of the explored section along with their relative orientation with respect to the fiber axis. The experiment was performed at 100 K to limit the degradation of the grain in the X-ray beam. (Taken from reference [91] with permission from Actualité Chimique http://www.lactualitechimique.org).

At the resolution of these ultra-structural features (10 µm) there is no discontinuity of orientation, i.e., no disclination of orientation. Between 10 µm steps, the change of the direction of the double-helices is gradual, which is consistent with a radial orientation (Figure 20) [92].

## Conclusion

The aim of the present article was to describe how synchrotron radiation has benefited the field of structural glycoscience in the studies of complex carbohydrates. The atomic structures of numerous (macro)-molecules have been revealed, from molecular single crystals all the way to the complexity of polysaccharide architectures, throughout the field of protein–carbohydrate interactions. Seemingly, the study of the less well explored area of colloids and glycolipids in their membrane environments can be tackled. The increasing speed of data collection times and photon flux are opening the way to time-resolved studies. The application of kinetic crystallography to elucidate glyco-enzymatic mechanisms is still at its infancy. Complementary to instrumental developments, the contribution of organic synthesis will be essential for the development of cage compounds, tailored to initiate light-activated reactions.

The results that have been presented were obtained on third-generation synchrotron sources. More sophisticated fourth-generation X-ray linear sources (X-ray Free Electron Lasers – XFEL) are operating at Stanford (USA), Hamburg (Germany) and in Harima (Japan). The brightness of the X-ray beams are then orders of magnitude greater and with short pulses, down to a few femtoseconds. A world full of novel experiments can be envisaged involving diffraction as well as the possibility to image non-periodic materials. Furthermore, different third-generation sources are planning major upgrades of their machine lattice to produce diffraction limited storage rings (DLSR) that will open new avenues in the science performed at these sources.

Synchrotron radiation offers much more than diffraction experiments and many other experiments can be performed making use of either the spectroscopy or imaging techniques. Spectroscopy techniques allow identification and characterization of molecular substances and their dynamics. Imaging techniques use the light-source beam to obtain pictures with spatial resolution of the sample under study. Integration of the results gathered from such experiments is a requirement to get deeper insight into the structures and mechanisms of vital biological processes in plants, animals and human.

## References

[1] Eriksson M, van der Veen J F, Quitmann C (2014). J Synchrotron Radiat.

[2] Bianconi A, Dell'Ariccia M, Durham P J, Pendry B (1982). Phys Rev B: Condens Matter Mater Phys.

[3] Bragg W H, Bragg W L (1913). Proc R Soc London, Ser A.

[4] Martinez-Criado G, Borfecchia E, Mino L, Lamberti C, Lamberti C, Agostini G (2013). Micro and Nano X-ray beams. Characterization of Semiconductor Heterostructures and Nanostructures.

[5] Riekel C (2000). Rep Prog Phys.

[6] Kirkpatrick P, Baez A V (1948). J Opt Soc Am.

[7] Snigirev A, Kohn V, Snigireva I, Lengeler B (1996). Nature.

[8] Hendrickson W A, Teeter M M (1981). Nature.

[9] Karle J (1980). Int J Quantum Chem.

[10] Fischer E (1891). Ber Dtsch Chem Ges.

[11] Raymond S, Heyraud A, Tran Qui D, Kvick A, Chanzy H (1995). Macromolecules.

[12] Rencurosi A, Mitchell E P, Cioci G, Pérez S, Pereda-Miranda R, Imberty A (2004). Angew Chem, Int Ed.

[13] Brunelli M, Wright J P, Vaughan G B M, Mora A J, Fitch A N (2003). Angew Chem, Int Ed.

[14] Ochsenbein P, Kieffer J, El Hajji M (2010). European Powder Diffraction Conference, Darmstadt, Germany.

[15] Collings I, Watier Y, Giffard M, Dagogo S, Kahn R, Bonneté F, Wright J P, Fitch A N, Margiolaki I (2010). Acta Crystallogr, Sect D: Biol Crystallogr.

[16] Karavassili F, Giannopoulou A E, Kotsiliti E, Knight L, Norrman M, Schluckebier G, Drube L, Fitch A N, Wright J P, Margiolaki I (2012). Acta Crystallogr, Sect D: Biol Crystallogr.

[17] Margiolaki I, Giannopoulou A E, Wright J P, Knight L, Norrman M, Schluckebier G, Fitch A N, Von Dreele R B (2013). Acta Crystallogr, Sect D: Biol Crystallogr.

[18] Rose P W, Prlić A, Altunkaya A, Bi C, Bradley A R, Christie C H, Di Costanzo L, Duarte J M, Dutta S, Feng Z (2017). Nucleic Acids Res.

[19] Stevens R C (2000). Curr Opin Struct Biol.

[20] Cipriani F, Röwer M, Landret C, Zander U, Felisaz F, Márquez J A (2012). Acta Crystallogr, Sect D: Biol Crystallogr.

[21] Flot D, Mairs T, Giraud T, Guijarro M, Lesourd M, Rey V, van Brussel D, Morawe C, Borel C, Hignette O (2010). J Synchrotron Radiat.

[22] de Sanctis D, Beteva A, Caserotto H, Dobias F, Gabadinho J, Giraud T, Gobbo A, Guijarro M, Lentini M, Lavault B (2012). J Synchrotron Radiat.

[23] Audfray A, Claudinon J, Abounit S, Ruvoën-Clouet N, Larson G, Smith D F, Wimmerová M, Le Pendu J, Römer W, Varrot A (2012). J Biol Chem.

[24] Gabadinho J, Beteva A, Guijarro M, Rey-Bakaikoa V, Spruce D, Bowler M W, Brockhauser S, Flot D, Gordon E J, Hall D R (2010). J Synchrotron Radiat.

[25] de Sanctis D, Oscarsson M, Popov A, Svensson O, Leonard G (2016). Acta Crystallogr, Sect D.

[26] Stepanov S, Makarov O, Hilgart M, Pothineni S B, Urakhchin A, Devarapalli S, Yoder D, Becker M, Ogata C, Sanishvili R (2011). Acta Crystallogr, Sect D: Biol Crystallogr.

[27] Delagenière S, Brenchereau P, Launer L, Ashton A W, Leal R, Veyrier S, Gabadinho J, Gordon E J, Jones S D, Levik K E (2011). Bioinformatics.

[28] Monaco S, Gordon E, Bowler M W, Delagenière S, Guijarro M, Spruce D, Svensson O, McSweeney S M, McCarthy A A, Leonard G (2013). J Appl Crystallogr.

[29] Bowler M W, Nurizzo D, Barrett R, Beteva A, Bodin M, Caserotto H, Delagenière S, Dobias F, Flot D, Giraud T (2015). J Synchrotron Radiat.

[30] Higgins S J (1999). Protein Expression: A Practical Approach.

[31] Garman S C, Wurzburg B A, Tarchevskaya S S, Kinet J-P, Jardetzky T S (2000). Nature.

[32] Lee J E, Fusco M L, Saphire E O (2009). Nat Protoc.

[33] Agirre J, Ariza A, Offen W A, Turkenburg J P, Roberts S M, McNicholas S, Harris P V, McBrayer B, Dohnalek J, Cowtan K D (2016). Acta Crystallogr, Sect D.

[34] Agirre J, Iglesias-Fernández J, Rovira C, Davies G J, Wilson K S, Cowtan K D (2015). Nat Struct Biol.

[35] Breton C, Fournel-Gigleux S, Palcic M M (2012). Curr Opin Struct Biol.

[36] Coutinho P M, Deleury E, Davies G J, Henrissat B (2003). J Mol Biol.

[37] Albesa-Jové D, Guerin M E (2016). Curr Opin Struct Biol.

[38] Pedersen L C, Dong J, Taniguchi F, Kitagawa H, Krahn J M, Pedersen L G, Sugahara K, Negishi M (2003). J Biol Chem.

[39] Hu Y, Chen L, Ha S, Gross B, Falcone B, Walker D, Mokhtarzadeh M, Walker S (2003). Proc Natl Acad Sci U S A.

[40] Rocha J, Cicéron F, de Sanctis D, Lelimousin M, Chazalet V, Lerouxel O, Breton C (2016). Plant Cell.

[41] Lombard V, Bernard T, Rancurel C, Brumer H, Coutinho P M, Henrissat B (2010). Biochem J.

[42] Henrissat B, Davies G (1997). Curr Opin Struct Biol.

[43] Boraston A B, Bolam D N, Gilbert H J, Davies G J (2004). Biochem J.

[44] Gilbert H J, Knox J P, Boraston A B (2013). Curr Opin Struct Biol.

[45] Pérez S, Sarkar A, Rivet A, Breton A, Imberty A, Lütteke T, Frank M (2015). Glyco3D: A Portal for Structural Glycosciences. Glycoinformatics.

[46] Somers W S, Tang J, Shaw G D, Camphausen R T (2000). Cell.

[47] Hamelryck T W, Loris R, Bouckaert J, Dao-Thi M-H, Strecker G, Imberty A, Fernandez E, Wyns L, Etzler M E (1999). J Mol Biol.

[48] Šulák O, Cioci G, Delia M, Lahmann M, Varrot A, Imberty A, Wimmerová M (2010). Structure.

[49] Blanchard B, Nurisso A, Hollville E, Tétaud C, Wiels J, Pokorná M, Wimmerová M, Varrot A, Imberty A (2008). J Mol Biol.

[50] Merritt E A, Kuhn P, Sarfaty S, Erbe J L, Holmes R K, Hol W G (1998). J Mol Biol.

[51] Kumar A, Sýkorová P, Demo G, Dobeš P, Hyršl P, Wimmerová M (2016). J Biol Chem.

[52] Hester G, Kaku H, Goldstein I J, Schubert Wright C (1995). Nat Struct Biol.

[53] Sartim M A, Pinheiro M P, de Pádua R A, Sampaio S V, Nonato M C (2017). Toxicon.

[54] Buts L, Loris R, De Genst E, Oscarson S, Lahmann M, Messens J, Brosens E, Wyns L, De Greve H, Bouckaert J (2003). Acta Crystallogr, Sect D: Biol Crystallogr.

[55] Kostlánová N, Mitchell E P, Lortat-Jacob H, Oscarson S, Lahmann M, Gilboa-Garber N, Chambat G, Wimmerová M, Imberty A (2005). J Biol Chem.

[56] Gallego del Sol F, Gómez J, Hoos S, Nagano C S, Cavada B S, England P, Calvete J J (2005). Acta Crystallogr, Sect F: Struct Biol Cryst Commun.

[57] Cioci G, Mitchell E P, Chazalet V, Debray H, Oscarson S, Lahmann M, Gautier C, Breton C, Pérez S, Imberty A (2006). J Mol Biol.

[58] Imberty A, Lortat-Jacob H, Pérez S (2007). Carbohydr Res.

[59] Li W, Johnson D J, Esmon C T, Huntington J A (2004). Nat Struct Mol Biol.

[60] Abramson J, Smirnova I, Kasho V, Verner G, Kaback H R, Iwata S (2003). Science.

[61] Deng D, Xu C, Sun P, Wu J, Yan C, Hu M, Yan N (2014). Nature.

[62] Bianchi L, Diez-Sampedro A (2010). PLoS One.

[63] Cao Y, Jin X, Levin E J, Huang H, Zong Y, Quick M, Weng J, Pan Y, Love J, Punta M (2011). Nature.

[64] Dang S, Sun L, Huang Y, Lu F, Liu Y, Gong H, Wang J, Yan N (2010). Nature.

[65] Faham S, Watanabe A, Besserer G M, Cascio D, Specht A, Hirayama B A, Wright E M, Abramson J (2008). Science.

[66] Kumar H, Kasho V, Smirnova I, Finer-Moore J S, Kaback H R, Stroud R M (2014). Proc Natl Acad Sci U S A.

[67] McCoy J G, Ren Z, Stanevich V, Lee J, Mitra S, Levin E J, Poget S, Quick M, Im W, Zhou M (2016). Structure.

[68] Oldham M L, Chen J (2011). Science.

[69] Sun L, Zeng X, Yan C, Sun X, Gong X, Rao Y, Yan N (2012). Nature.

[70] Wang J, Yan C, Li Y, Hirata K, Yamamoto M, Yan N, Hu Q (2014). Cell Res.

[71] Watanabe A, Choe S, Chaptal V, Rosenberg J M, Wright E M, Grabe M, Abramson J (2010). Nature.

[72] Xu Y, Tao Y, Cheung L S, Fan C, Chen L Q, Xu S, Perry K, Frommer W B, Feng L (2014). Nature.

[73] Nomura N, Verdon G, Kang H J, Shimamura T, Nomura Y, Sonoda Y, Hussien S A, Qureshi A A, Coincon M, Sato Y (2015). Nature.

[74] Bourgeois D, Weik M (2009). Crystallogr Rev.

[75] Batot G (2013). Une nouvelle approche pour étudier le mécanisme des glycosyltransférases.

[76] Jørgensen R, Batot G, Mannerstedt K, Imberty A, Breton C, Hindsgaul O, Royant A, Palcic M M (2014). Acta Crystallogr, Sect F: Struct Biol Cryst Commun.

[77] Menchicchi B, Fuenzalida J P, Hensel A, Swamy M J, David L, Rochas C, Goycoolea F M (2015). Biomacromolecules.

[78] Pernot P, Round A, Barrett R, De Maria Antolinos A, Gobbo A, Gordon E, Huet J, Kieffer J, Lentini M, Mattenet M (2013). J Synchrotron Radiat.

[79] Round A, Felisaz F, Fodinger L, Gobbo A, Huet J, Villard C, Blanchet C E, Pernot P, McSweeney S, Roessle M (2015). Acta Crystallogr, Sect D: Biol Crystallogr.

[80] Bizien T, Durand D, Roblina P, Thureau A, Vachette P, Pérez J (2016). Protein Pept Lett.

[81] De Maria Antolinos A, Pernot P, Brennich M E, Kieffer J, Bowler M W, Delageniere S, Ohlsson S, Malbet Monaco S, Ashton A, Franke D (2015). Acta Crystallogr, Sect D: Biol Crystallogr.

[82] Daillant J, Gibaud A (1999). X-ray and Neutron Reflectivity: Principles and Applications.

[83] Rondelli V, Brocca P, Fragneto G, Daillant J, Tringali C, Cantu C, Del Favero E (2017). Biochim Biophys Acta, Biomembr.

[84] Nishiyama Y, Sugiyama J, Chanzy H, Langan P (2003). J Am Chem Soc.

[85] Nishiyama Y, Langan P, Chanzy H (2002). J Am Chem Soc.

[86] Imberty A, Chanzy H, Pérez S, Buléon A, Tran V (1988). J Mol Biol.

[87] Imberty A, Pérez S (1988). Biopolymers.

[88] Popov D, Buléon A, Burghammer M, Chanzy H, Montesanti N, Putaux J-L, Potocki-Véronèse G, Riekel C (2009). Macromolecules.

[89] Lichtenegger H, Müller M, Paris O, Riekel C, Fratzl P (1999). J Appl Crystallogr.

[90] Hsiao B S (2015). Structure characterization of cellulose nanofibers-microfibrills. ALBA Synchrotron - Marie Curie Meeting Room.

[91] Pérez S, Burghammer M (2011). Actual Chim.

[92] Chanzy H, Putaux J-L, Dupeyre D, Davies R, Burghammer M, Montanari S, Riekel C (2006). J Struct Biol.

